# Supplementary figures and images for: Cross-species regulatory sequence activity prediction
Source: PLoS Comput Biol. 2020 Jul 20;16(7):e1008050. doi: 10.1371/journal.pcbi.1008050 (PMC7392335; doi:10.1371/journal.pcbi.1008050)

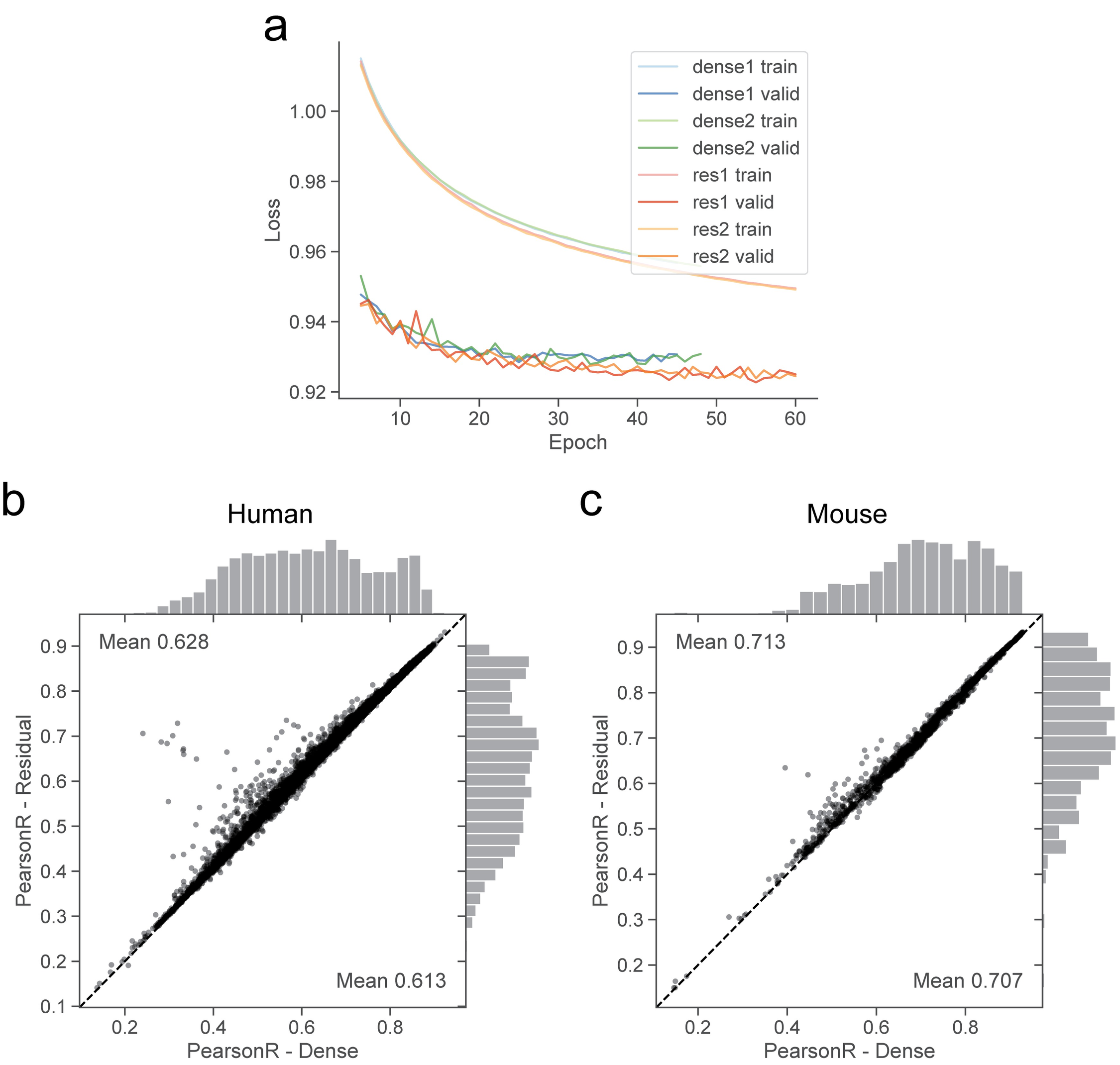

Supplement: S1 Fig — We trained two separate models with approximately matched parameter totals on both human and mouse data jointly. The two models differ in how their dilated convolution layers are connected. In the first model Dense, which achieved the previous best for these data in [5], each layer takes all previous dilated layers as input, as opposed to taking only the preceding layer. In the second model Residual, introduced here, each layer takes only the previous layer as input, transforms it, and adds the new representation into the input before passing on. For each model, we computed the Pearson correlation of test set predictions and observed experimental data for thousands of datasets from various experiment types. (a) Training and validation loss curves for two replicates of the two architectures with random initializations and shuffled training examples. The top curves represent the training set and the bottom curves represent the validation set. Validation losses are less than training losses due only to stochasticity in the sequence splitting procedure. (b) Human and (c) mouse test set Pearson correlation for the best Dense versus Residual model. (TIF) [file pcbi.1008050.s001.tif]

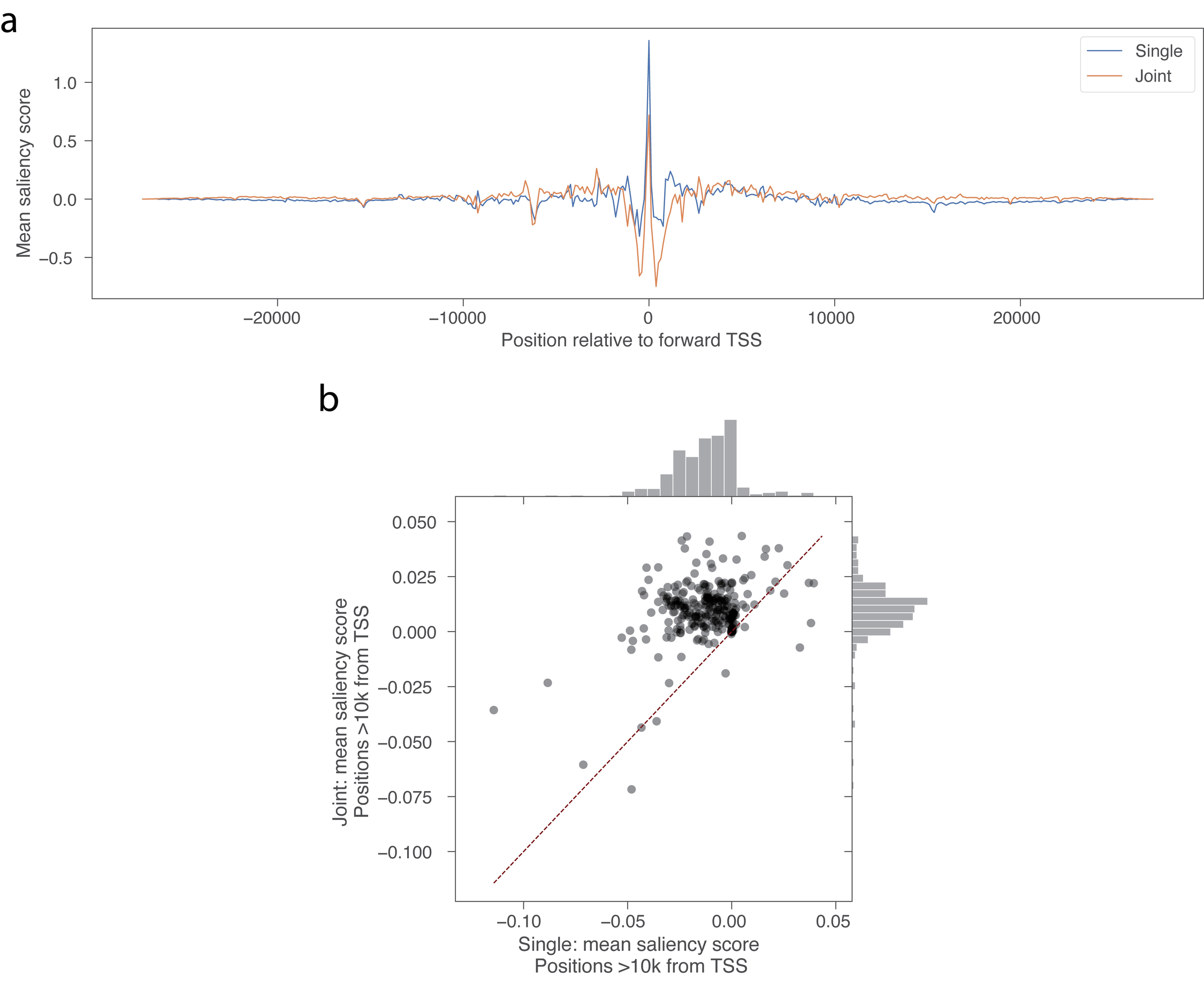

Supplement: S2 Fig — For 3,523 gene transcription start sites (TSS) that were not included in the training set, we computed saliency maps for the surrounding region using the model trained jointly on human and mouse (joint) and the model trained on human alone (single). The saliency map scores annotate 128 bp segments with a function of the model predictions’ gradient with respect to that segment’s vector representation after the convolutional layers and before the dilated convolutions share information across wider distances [5]. Peaks in this saliency score detect distal regulatory elements, and its sign indicates enhancing (+) versus repressing (−) influence. (a) For each 128-bp segment, we computed the mean score across genes for liver CAGE. Patterns were consistent across CAGE datasets. (b) For segments greater than 10 kb from the TSS, the mean multi-genome model scores are greater than their single genome counterparts. This suggests that distal enhancer elements are more effectively used to predict gene expression. (TIF) [file pcbi.1008050.s002.tif]

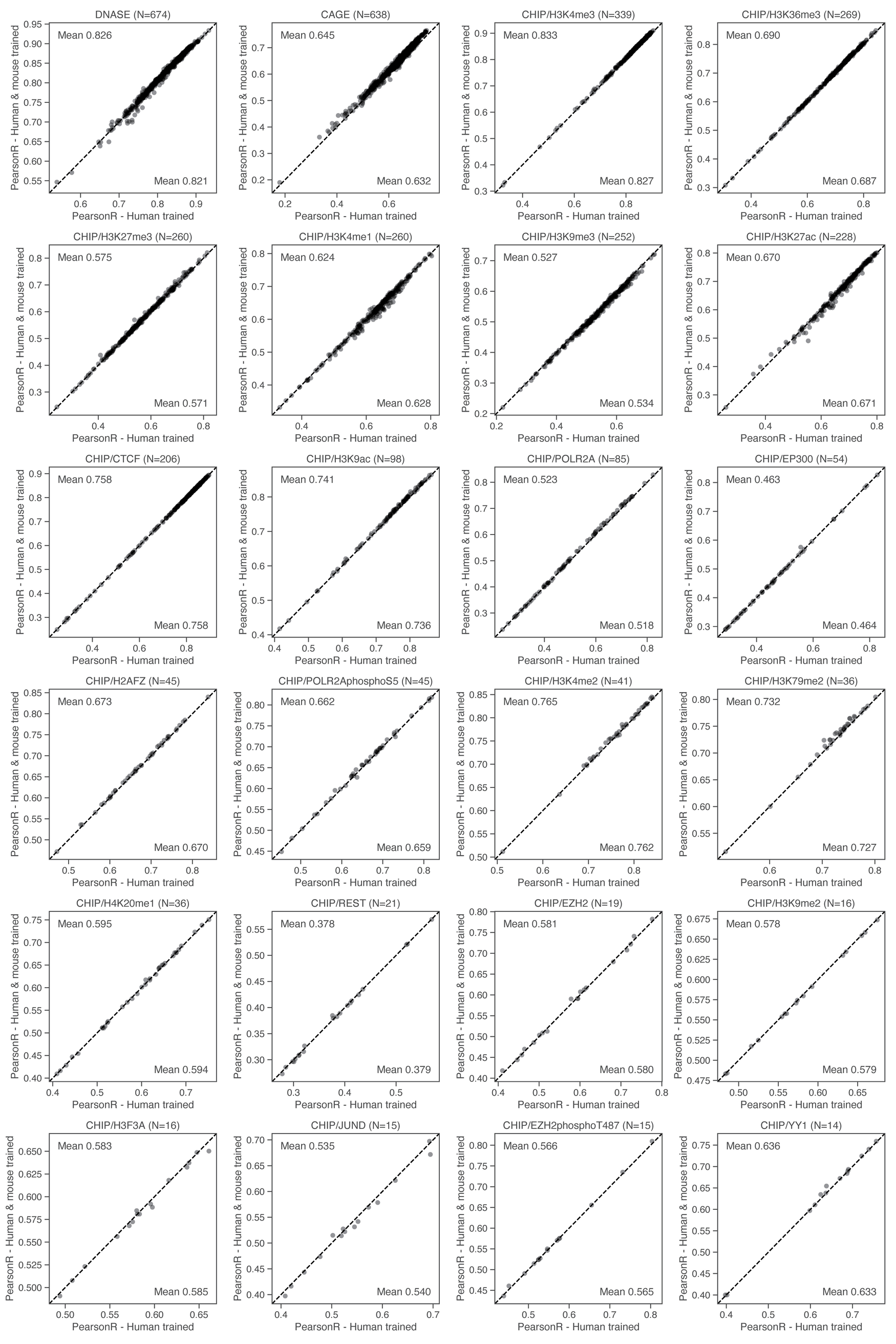

Supplement: S3 Fig — We trained the same architecture on human data alone and both human and mouse data jointly. For each model, we computed the Pearson correlation of test set predictions and observed experimental data for human CAGE, DNase, and ChIP-seq datasets. Points in the scatter plots represent individual datasets, with single genome training accuracy on the x-axis and joint training accuracy on the y-axis. We considered these accuracy comparisons, broken down by experiment class for the 24 most frequent experiments. Within the various categories, the improvement differed slightly. For example, H3K4me3 increased by 0.006, but H3K9me3 decreased by 0.007. Enhancer marks H3K4me1 (-0.004), H3K27ac (0), and P300 (-0.001) were stable or unimproved. (TIF) [file pcbi.1008050.s003.tif]

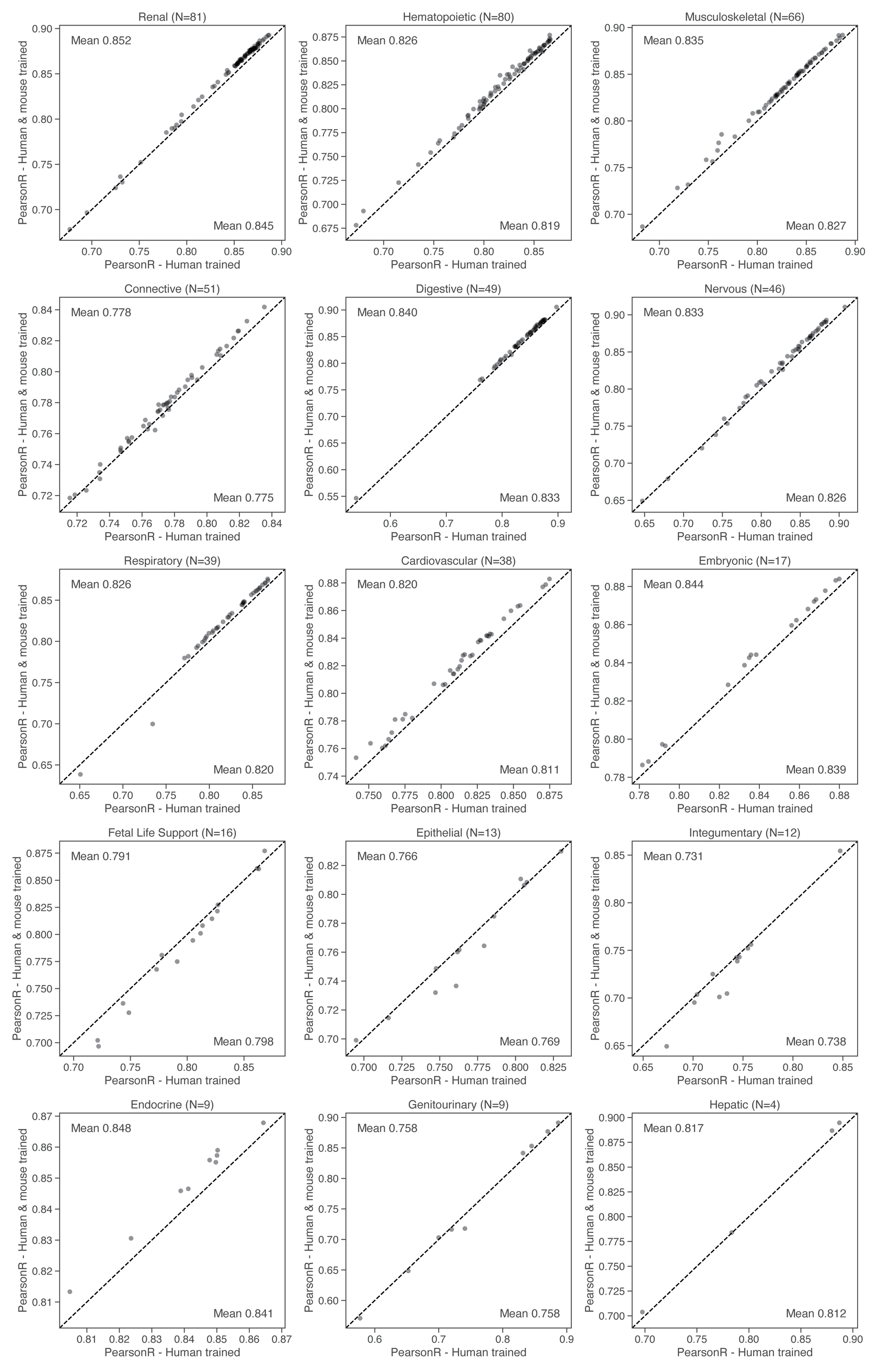

Supplement: S4 Fig — We trained the same architecture on human data alone and both human and mouse data jointly. For each model, we computed the Pearson correlation of test set predictions and observed experimental data for human DNase datasets. Points in the scatter plots represent individual datasets, with single genome training accuracy on the x-axis and joint training accuracy on the y-axis. We considered these accuracy comparisons in the context of fifteen organ system annotations assigned by Meuleman et al. [26]. Pearson R improves by an average of 0.006 across all of these DNase datasets. Within the various categories the improvement differed slightly. “Musculoskeletal” datasets improved by 0.008, which was significantly greater than the remainder by Mann-Whitney U test with p-value 2e − 7. In contrast, “Connective” datasets improved by 0.004, which was significantly less than the remainder with p-value 1e − 10. We were unable to discern a pattern that provided insight into why some categories improve more or less than others with joint training. (TIF) [file pcbi.1008050.s004.tif]

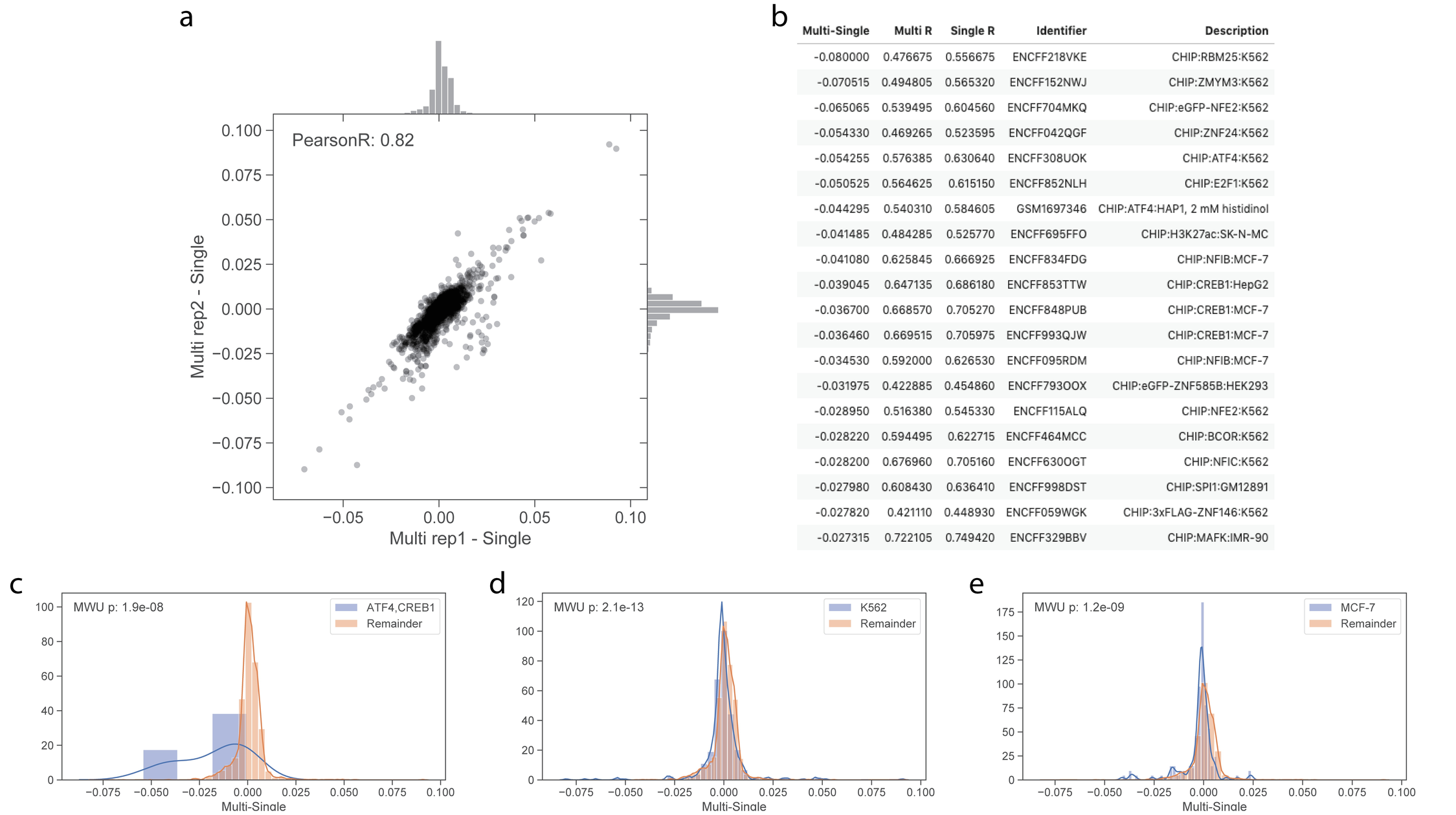

Supplement: S5 Fig — To further explore human ChIP-seq datasets that performed worse during multi-genome training relative to single genome training, we trained two independent replicates for both training modes. For the single genome training, we took the average of the two replicates. (a) For the multiple genome training, we plot the ChIP-seq test set PearsonR for replicate 1 minus the single genome PearsonR versus that for replicate 2. Dataset accuracy was consistent across replicates, and some ChIP-seq datasets consistently achieved lower accuracy after multi-genome training. (b) The table displays the 20 datasets with the largest decrease in test set accuracy after multi-genome training. Datasets describing (a) ATF4/CREB binding (known co-factors), (b) K562 cells, and (c) MCF-7 cells performed significantly worse according to Mann-Whitney U comparisons of the sets of 16, 476, and 129 datasets respectively. Histograms consider the average of test set PearsonR for multi-genome training minus the average for single genome training. (TIF) [file pcbi.1008050.s005.tif]

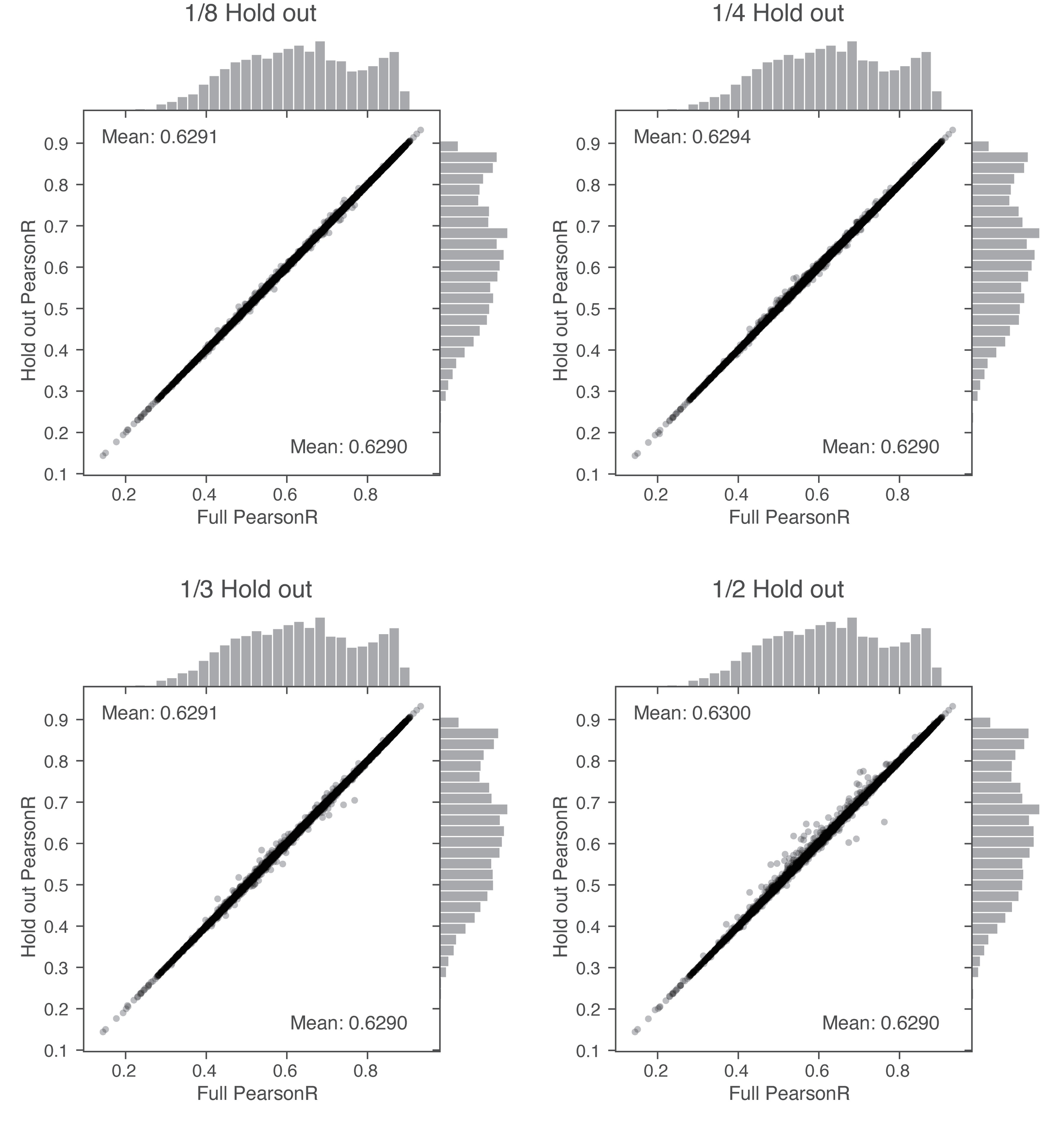

Supplement: S6 Fig — We designed several experiments to explore whether more datasets alone improve the models, rather than the datasets and novel sequence offered by the mouse genome. We split all human datasets into eight, four, three, or two folds. For each fold, we held out those datasets and trained a model only on the remainder. We trained four replicate models from random initializations on the full data for comparison. For the three fold experiment, we repeated the procedure with a unique random split twice. For the two fold experiment, we repeated the procedure with a unique random split three times. For each dataset, we averaged test set accuracy for the training runs that did train on it, each of which had a different portion of the datasets held out. Above, we scatter plot average test set accuracy for each of the 5,313 datasets for the full data versus the held out data runs for each experiment. If it were true that adding more annotations benefited model training and accuracy, then these models would suffer from the held out targets and show reduced accuracy relative to the full model. Instead, for each experiment with hold outs up to half of the datasets, these models achieved slightly greater mean accuracy than the full data model. (TIF) [file pcbi.1008050.s006.tif]

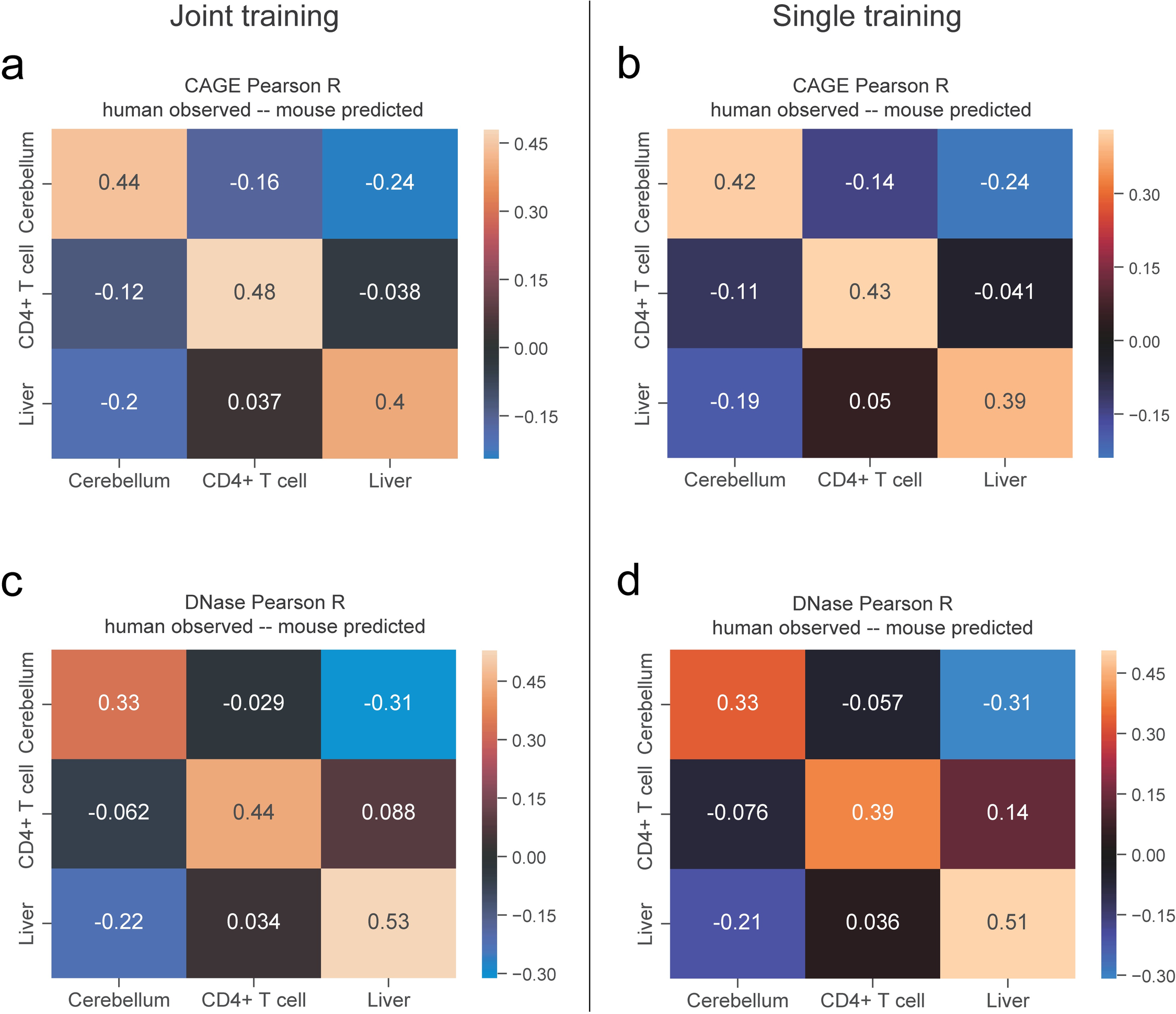

Supplement: S7 Fig — Tissue-specific regulatory programs can be learned and transferred across species, exemplified here by mouse predictions for CAGE (top row) and DNase (bottom row) for cerebellum, liver, and CD4+ T cells. Mouse predictions correspond to mouse datasets matched and compared to human datasets For CAGE, we considered the top 50% most variable TSSs, where data or predictions were quantile normalized to align sample distributions, log transformed, and mean-normalized across samples. For DNase, we considered the top 10% most variable genomic sites (less than CAGE because we consider the whole genome rather than TSSs), where data or predictions were similarly were quantile normalized to align sample distributions and mean-normalized across samples. The statistical trends were robust to most variable threshold choice. Tissue-specific cross-species accuracy depends only slightly whether the mouse model was trained jointly with human data (left column) or alone (right column). This is expected, given that the multi-genome model is more accurate on held out sequences (Fig 2). (TIF) [file pcbi.1008050.s007.tif]

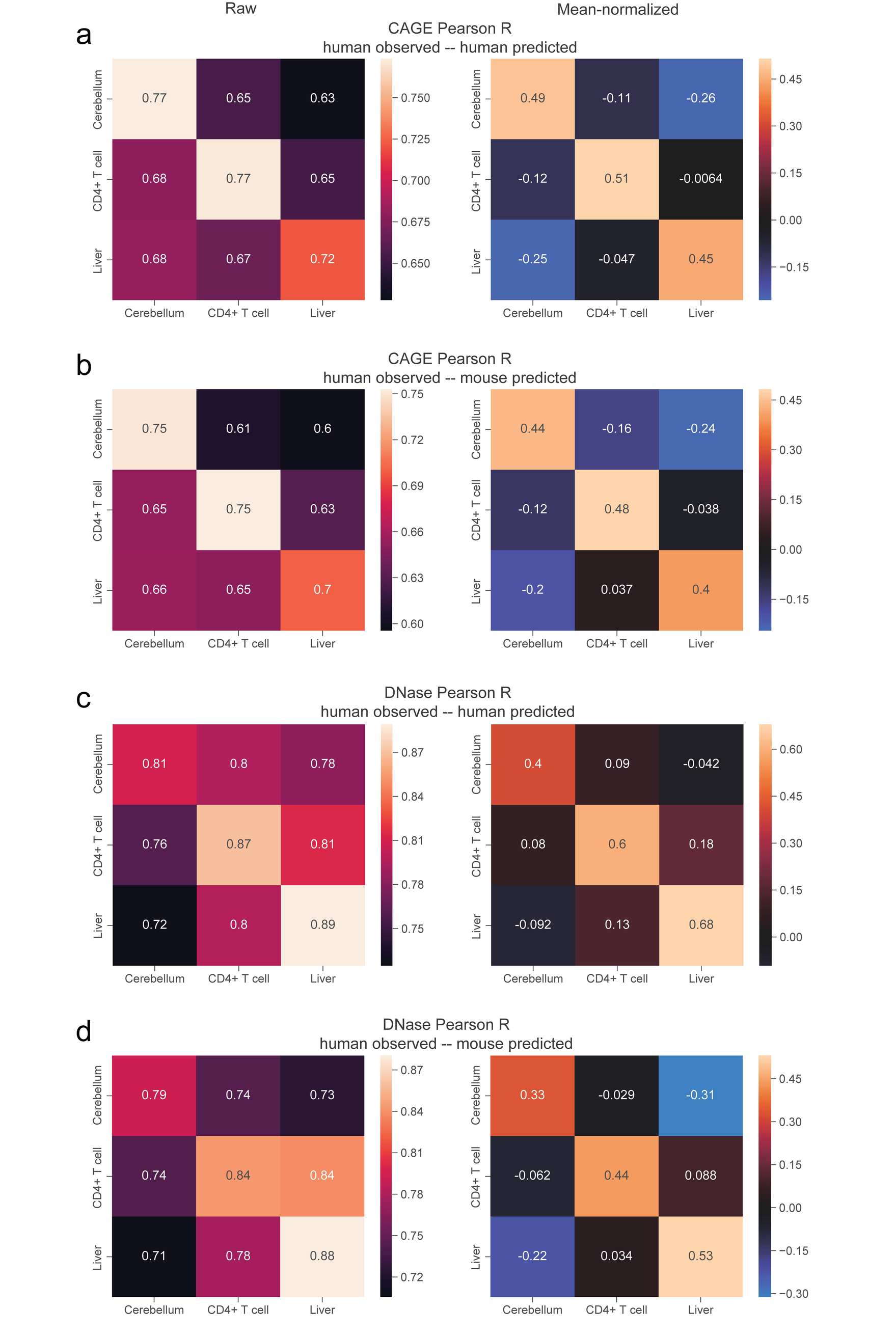

Supplement: S8 Fig — Tissue-specific regulatory programs can be learned and transferred across species, exemplified here by mouse predictions for CAGE (a,b) and DNase (c,d) for cerebellum, liver, and CD4+ T cells. “Human predicted” corresponds to predictions for the human datasets, referred to as “human observed”; “mouse predicted” corresponds to predictions for the matched mouse dataset. For CAGE, we considered the top 50% most variable TSSs, where data or predictions were quantile normalized to align sample distributions, and log transformed. In the right column, we mean-normalized across samples; in the left, we did not. For DNase, we considered the top 10% most variable genomic sites (less than CAGE because we consider the whole genome rather than TSSs), where data or predictions were similarly quantile normalized to align sample distributions and mean-normalized across samples in the right column only. The statistical trends were robust to most variable threshold choice. (a,c) Human prediction accuracies exceed (b,d) mouse prediction accuracies for both CAGE and DNase. (TIF) [file pcbi.1008050.s008.tif]

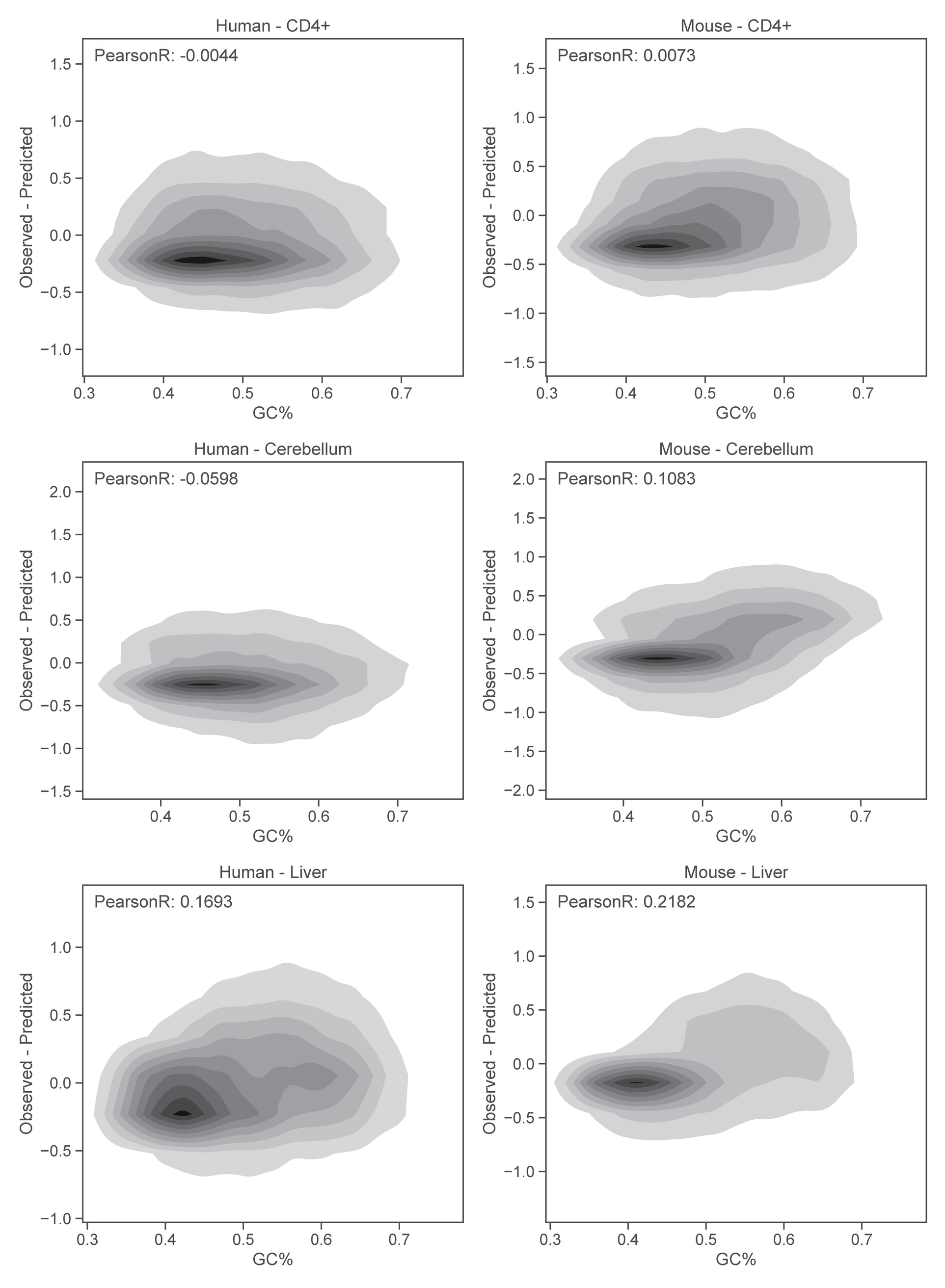

Supplement: S9 Fig — For the DNase sites studied across species, we computed residuals as the mean-normalized observed signal minus predicted signal. We computed GC% in a 1,000 bp region around the 128 bp segment. Correlations between GC content and the residuals were larger for mouse than human, indicating that mouse predictions may be slightly mis-calibrated for the human genome. (TIF) [file pcbi.1008050.s009.tif]

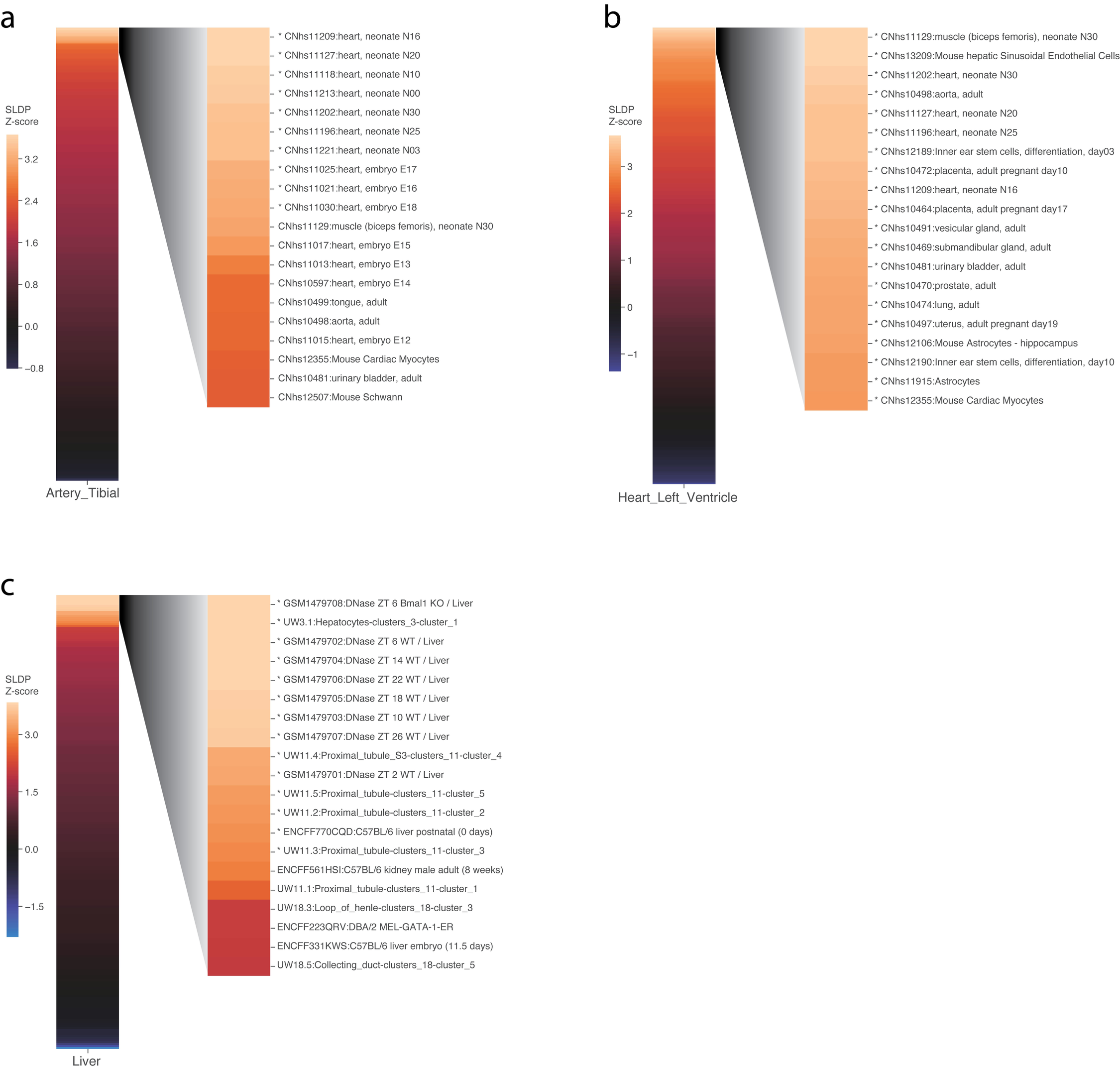

Supplement: S10 Fig — We computed variant effect predictions for all 1000 Genomes variants with respect to human and mouse datasets. We then analyzed the CAGE and DNase/ATAC data separately. We computed the first 64 principal components (PCs) of the variants by human predictions matrix, which explained 99.9% of the variance for CAGE datasets and 99.3% for DNase/ATAC. We then computed the statistical correlation between mouse predictions and GTEx summary statistics across 48 tissues using SLDP conditioned on the 64 human PCs for either the CAGE or DNase/ATAC data (Methods). (a) For the tibial artery and (c) left ventricle GTEx summary statistics, mouse CAGE datasets describing the developing heart in neonate and embryo stages emerged as significant after Benjamini–Hochberg correction for multiple hypotheses. Prefix asterisks indicate FDR q < 0.05. Additional datasets describing adult heart components and muscle also reach significance. (b) For liver GTEx, mouse single cell hepatocyte and DNase datasets describing a 24 hour time course to profile circadian rhythms of genome accessibility reach significance. (TIF) [file pcbi.1008050.s010.tif]

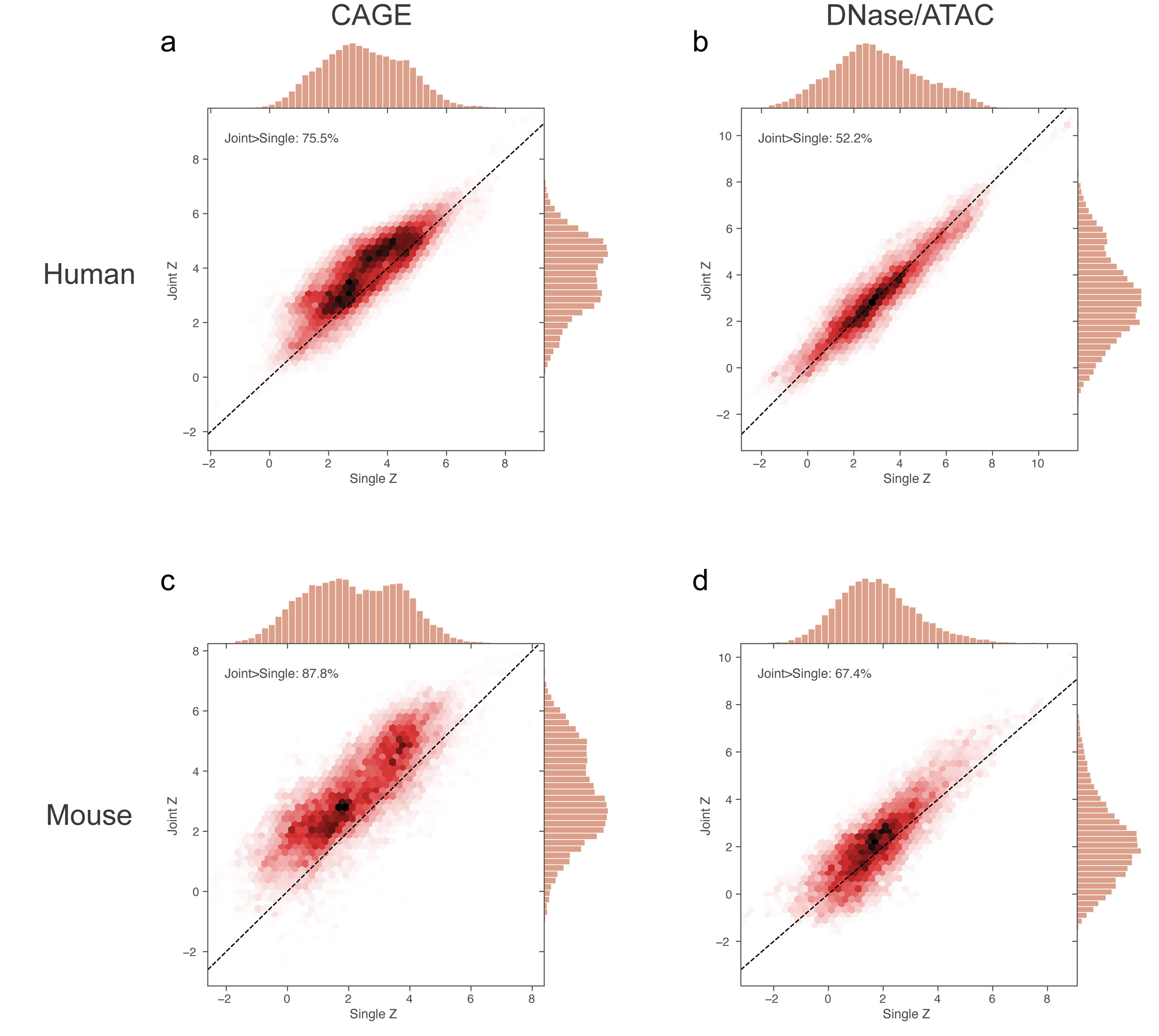

Supplement: S11 Fig — We computed variant effect predictions for all 1000 Genomes variants with respect to human and mouse datasets using models trained jointly on both human and mouse or trained alone on a single genome. We then computed the statistical correlation between these predictions and GTEx summary statistics across 48 tissues using SLDP (Methods). The points underlying the density maps represent every pair of model prediction dataset and GTEx tissue. SLDP signed Z-scores indicate the expected positive statistical relationship between predictions for CAGE, DNase, and ATAC-seq and gene expression. These Z-scores are clearly greater for predictions from jointly trained models for (a) human CAGE, (c) mouse CAGE, and (d) mouse DNAase/ATAC. (b) Human DNase/ATAC Z-scores are more similar between the joint and single trained models, in line with their comparable accuracy on held out sequences (Fig 2). (TIF) [file pcbi.1008050.s011.tif]

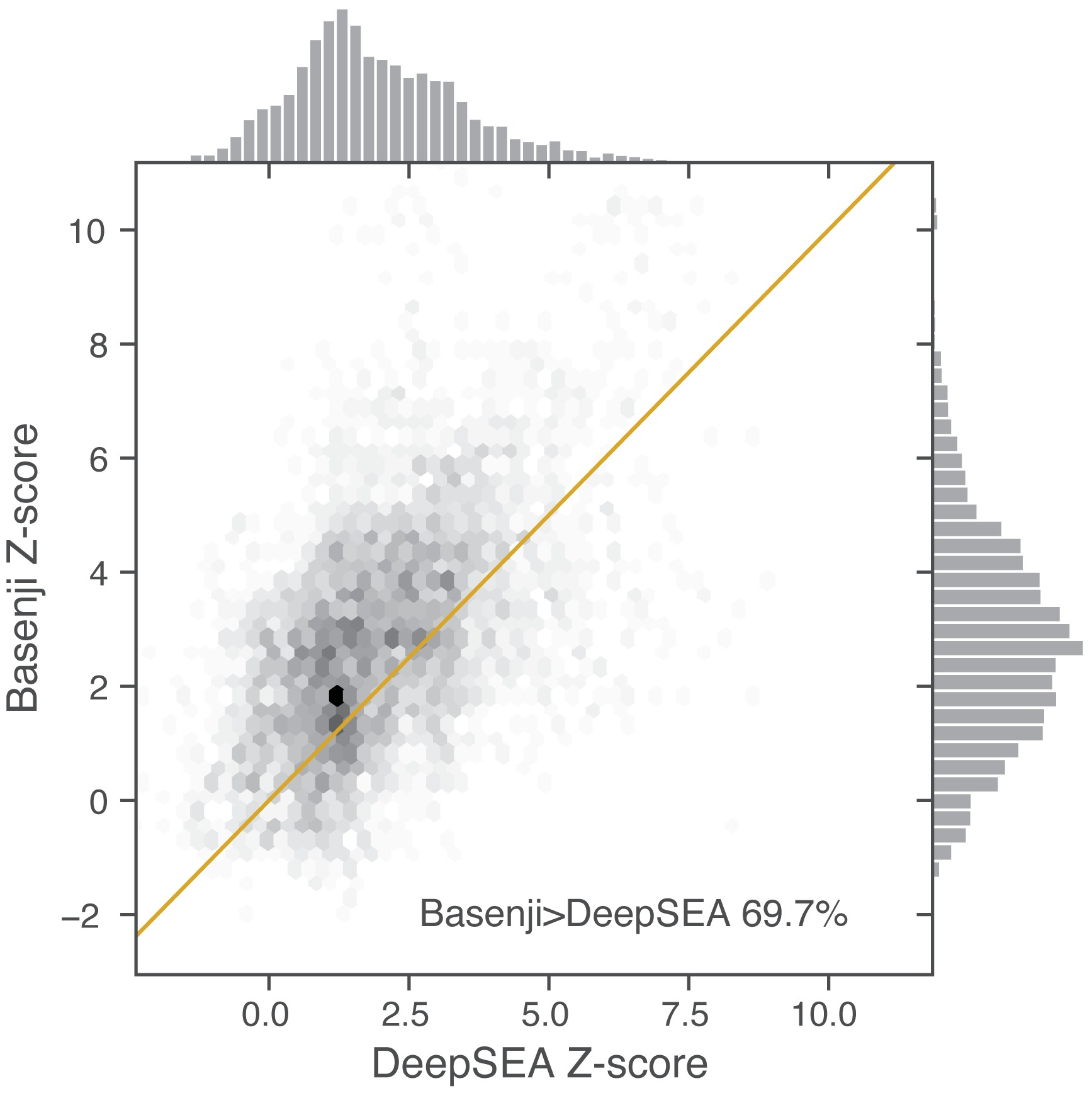

Supplement: S12 Fig — We computed variant effect predictions for all 1000 Genomes variants using the DeepSEA “beluga” model. We then computed the statistical correlation between these predictions and GTEx summary statistics across 48 tissues using SLDP (Methods). We manually aligned all human DNase datasets between the DeepSEA and Basenji models, arriving at 100 matched datasets. Here, we scatter plot Basenji versus DeepSEA Z-scores for each combination of DNAse dataset and GTEx tissue. Basenji Z-scores are greater for 69.7% of combinations (permuting Basenji/DeepSEA labels p-value <1 × 10^−9^). (TIF) [file pcbi.1008050.s012.tif]

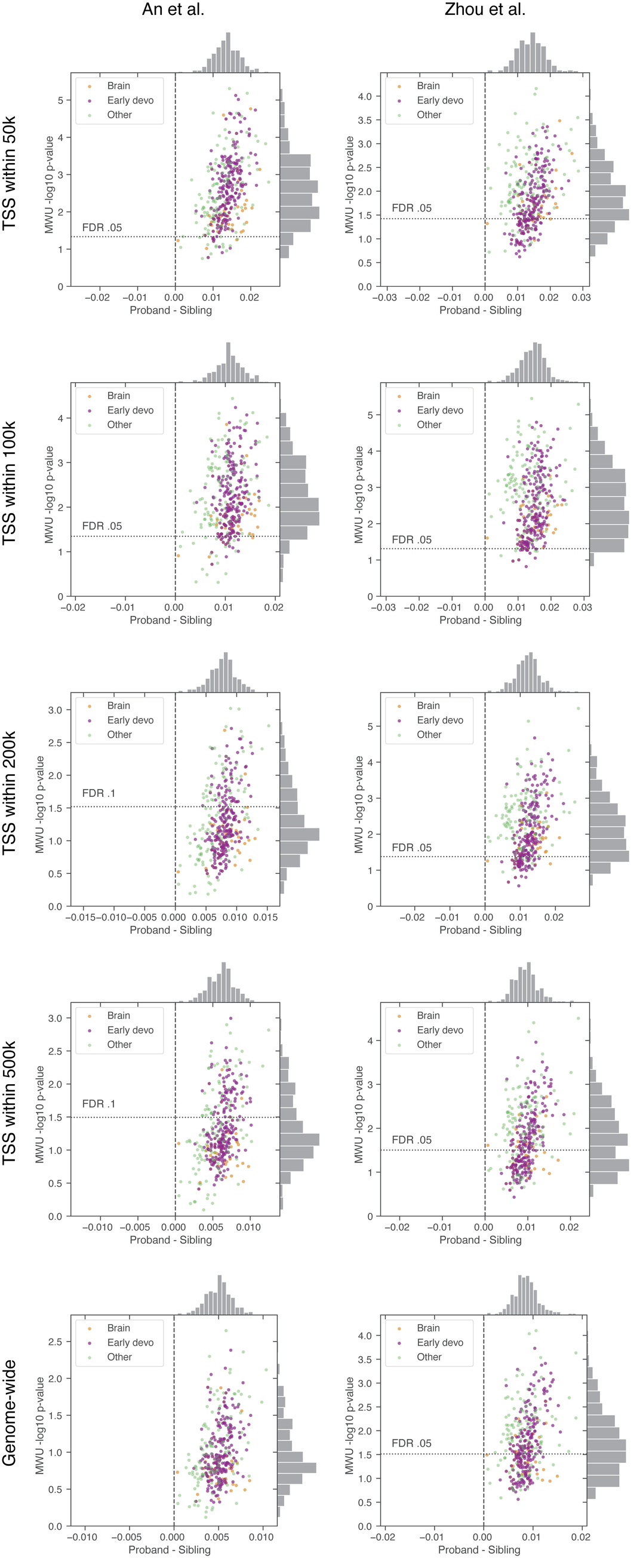

Supplement: S13 Fig — We predicted the influence of de novo variants found in proband and sibling genomes for mouse CAGE datasets. We studied two versions of the processed whole genome sequences by An et al. (left column) and Zhou et al. (right column) [43, 44]. In each plot, points represent CAGE datasets, from which a predicted activity difference can be assigned to each variant. We scaled negative variant scores by 10 before taking the absolute value. In each row, we filtered for variants within the specified distance of a GENOCODE mRNA TSS. On the x-axis, we plot the mean natural log score for all proband variants subtracted by the mean natural log score for all sibling variants. On the y-axis, we plot the log10 p-value from comparing proband to sibling scores using a Mann-Whitney U test. Variants nearby TSS contain more signal than those that are very far from genes, which may nonetheless have nonzero CAGE predictions due to enhancer RNA activity. At the most strict distance threshold of 30kb, the x-axis difference between probands and siblings continues to grow, but the y-axis significance is decreased by the smaller number of remaining variants. The Zhou et al. datasets are robust to the filter distance, but the An et al. datasets only reach FDR q-values < 0.1 with TSS filters < 500 kb (which keeps 90% of variants) and decrease as the filter distance tightens around TSS. (TIF) [file pcbi.1008050.s013.tif]

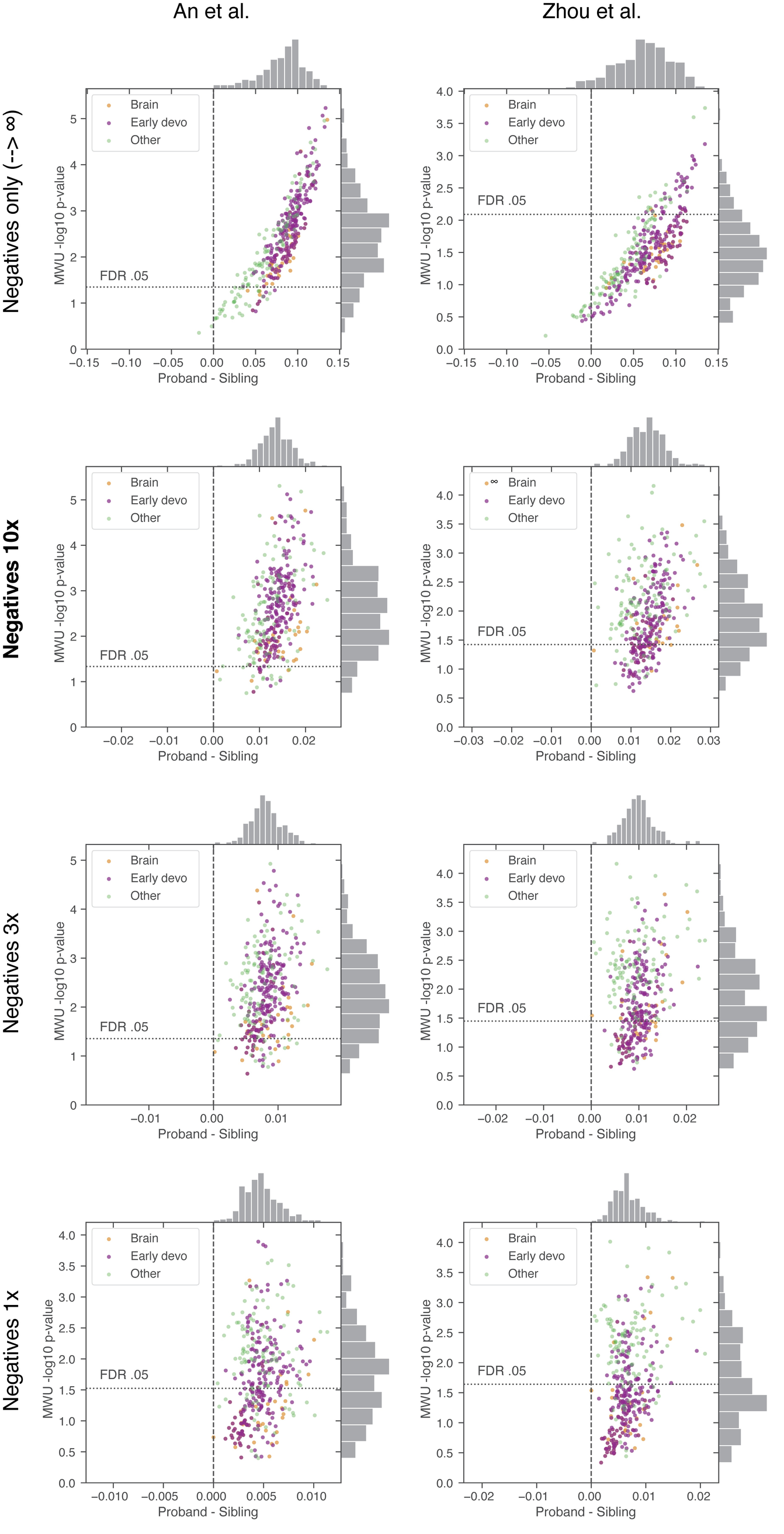

Supplement: S14 Fig — We predicted the influence of de novo variants found in proband and sibling genomes for mouse CAGE datasets. We studied two versions of the processed whole genome sequences by An et al. (left column) and Zhou et al. (right column) [43, 44]. In each plot, points represent CAGE datasets, from which a predicted activity difference can be assigned to each variant. We focused on variants within 50 kb of a GENCODE mRNA TSS. In each row, we scaled negative variant scores by the specified factor, before taking the absolute value. On the x-axis, we plot the mean natural log score for all proband variants subtracted by the mean natural log score for all sibling variants. On the y-axis, we plot the log10 p-value from comparing proband to sibling scores using a Mann-Whitney U test. The first row displays the statistical tests computed for only negative values, which represents the limit as the negative prediction weight grows to infinity. Negative predictions are more informative than positive, so an even weighting of the two produces less significant differentiation between the probands and their sibling controls. However, the results are very robust to the choice of the scaling factor. (TIF) [file pcbi.1008050.s014.tif]

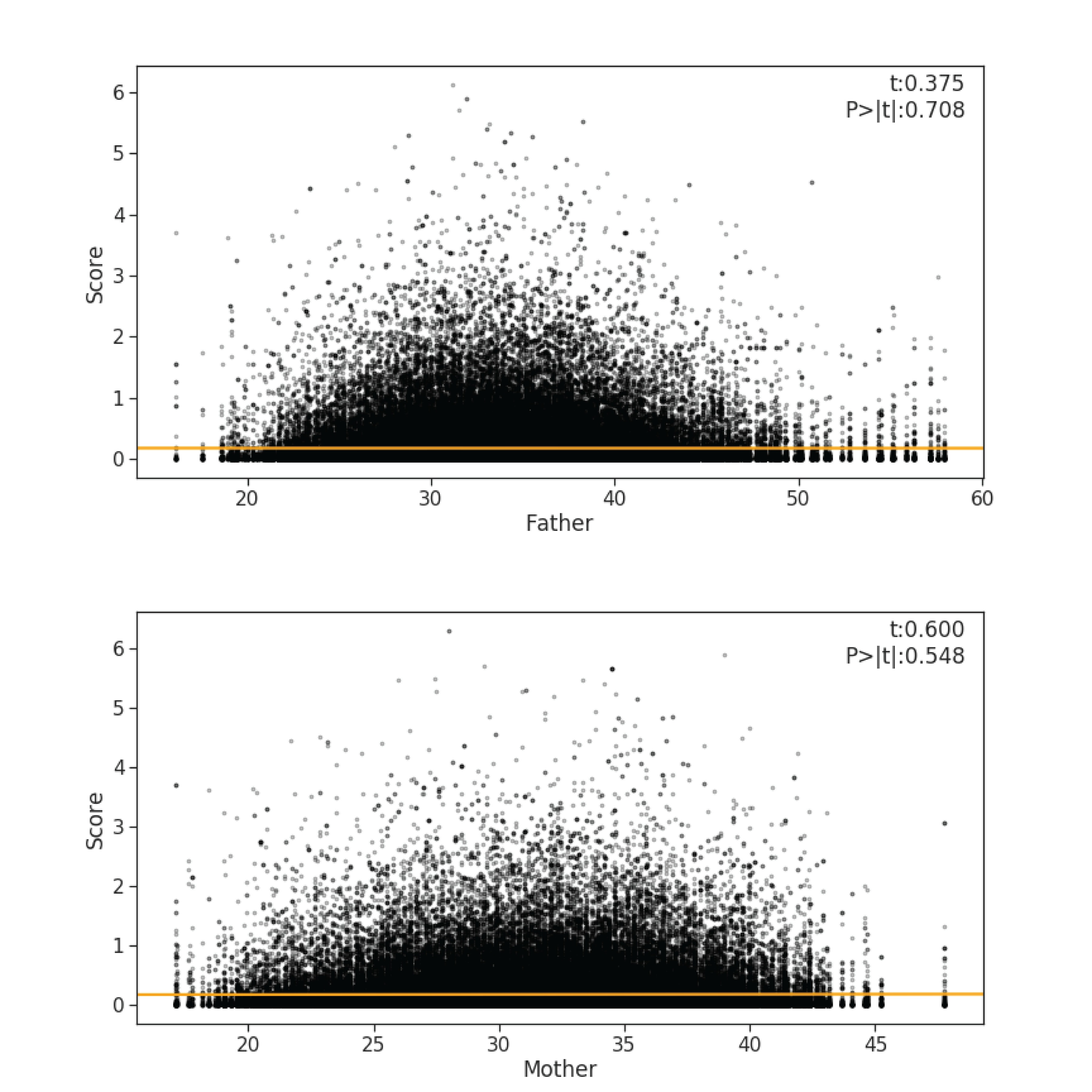

Supplement: S15 Fig — We predicted the influence of de novo variants found in proband and sibling whole genomes sequences processed by An et al. for mouse datasets [43]. Variant predictions are derived from a representative dataset of “whole body, embryo E16” profiled by CAGE that has significantly greater effect score for probands relative to their sibling controls. We transformed the raw variant predictions similarly to the main analyses by scaling negative variant scores by 10 before taking the absolute value and further adding a pseudocount of one and taking the natural logarithm to improve the stability of the scores for the visualization. Regression analysis indicates no dependency of the scores on the mother or father’s age at birth. This result matches that of Zhou et al. in their analysis of a separate processing of these data. [44]. (TIF) [file pcbi.1008050.s015.tif]

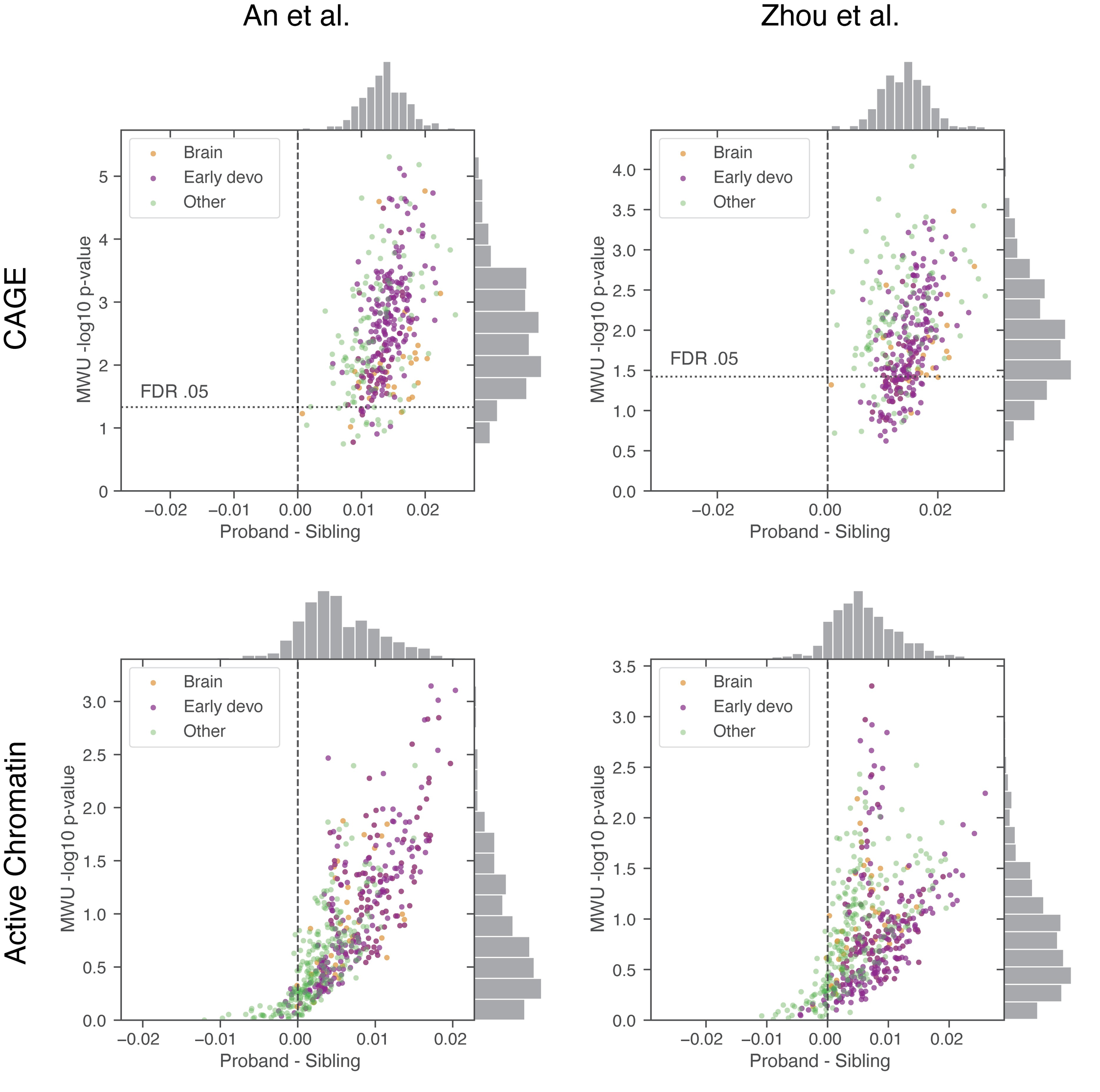

Supplement: S16 Fig — We predicted the influence of de novo variants found in proband and sibling genomes for mouse datasets. We studied two versions of the processed whole genome sequences by An et al. (left column) and Zhou et al. (right column) [43, 44]. We separated CAGE gene expression (top row) from active chromatin modifications DNase/ATAC/H3K4me3/H3K4me1/H3K27ac (bottom row). In each plot, points represent datasets, from which a predicted activity difference can be assigned to each variant. We focused on variants within 50 kb of a GENCODE mRNA TSS and scaled negative variant scores by 10 before taking the absolute value. On the x-axis, we plot the mean natural log score for all proband variants subtracted by the mean natural log score for all sibling variants. On the y-axis, we plot the log10 p-value from comparing proband to sibling scores using a Mann-Whitney U test. Many CAGE datasets show Benjamini-Hochberg q-values < 0.05; chromatin datasets demonstrate a similar trend toward greater scores for proband variants, but do not reach the same significance levels. (TIF) [file pcbi.1008050.s016.tif]

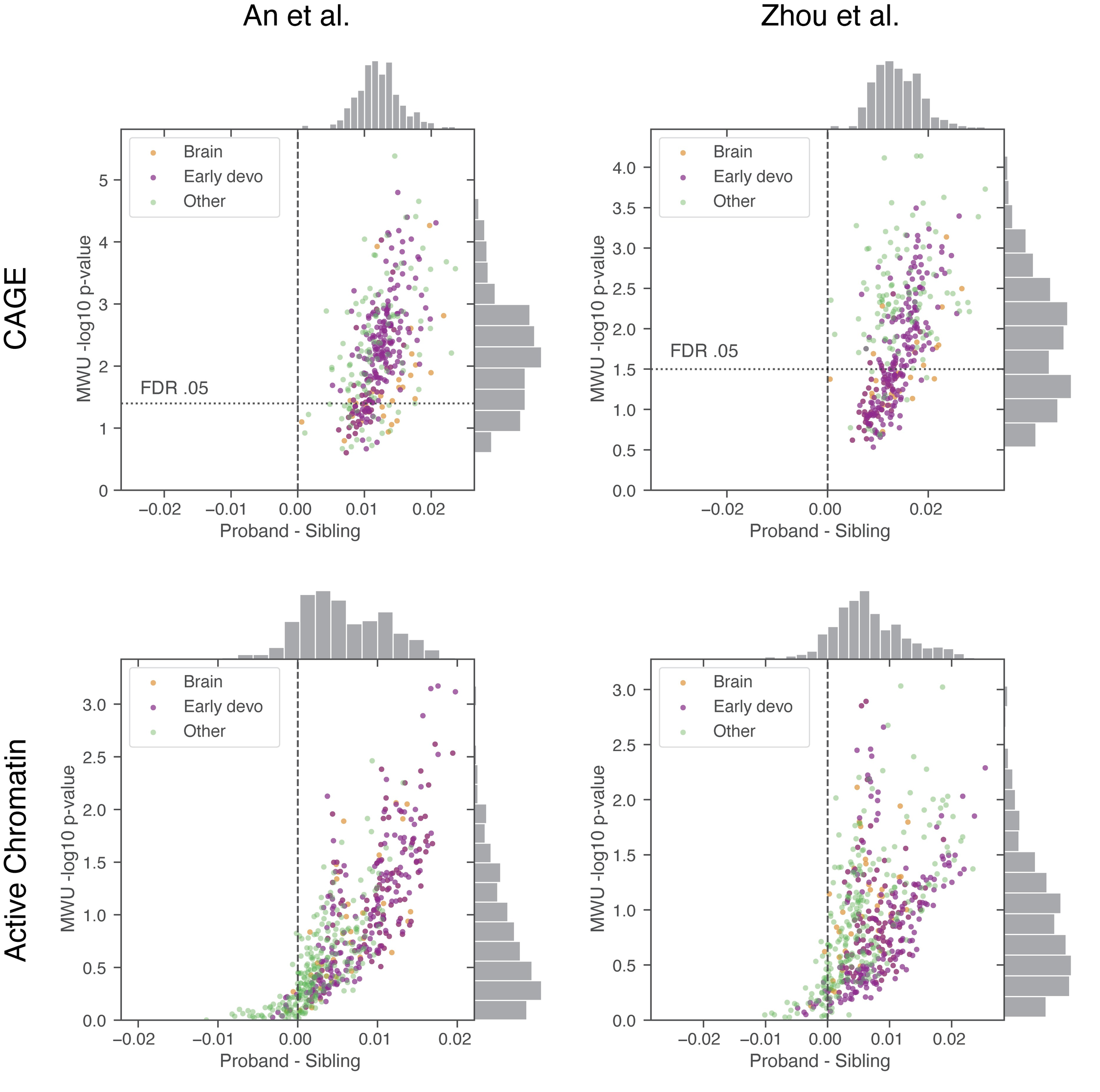

Supplement: S17 Fig — We predicted the influence of de novo variants found in proband and sibling genomes for mouse datasets. We studied two versions of the processed whole genome sequences by An et al. (left column) and Zhou et al. (right column) [43, 44]. We removed any variant overlapping GENCODE coding sequence, 1.7% and 3.3% respectively. We separated CAGE gene expression (top row) from active chromatin modifications DNase/ATAC/H3K4me3/H3K4me1/H3K27ac (bottom row). In each plot, points represent datasets, from which a predicted activity difference can be assigned to each variant. We focused on variants within 50 kb of a GENCODE mRNA TSS and scaled negative variant scores by 10 before taking the absolute value. On the x-axis, we plot the mean natural log score for all proband variants subtracted by the mean natural log score for all sibling variants. On the y-axis, we plot the log10 p-value from comparing proband to sibling scores using a Mann-Whitney U test. Many CAGE datasets show Benjamini-Hochberg q-values < 0.05; chromatin datasets demonstrate a similar trend toward greater scores for proband variants, but do not reach the same significance levels. (TIF) [file pcbi.1008050.s017.tif]

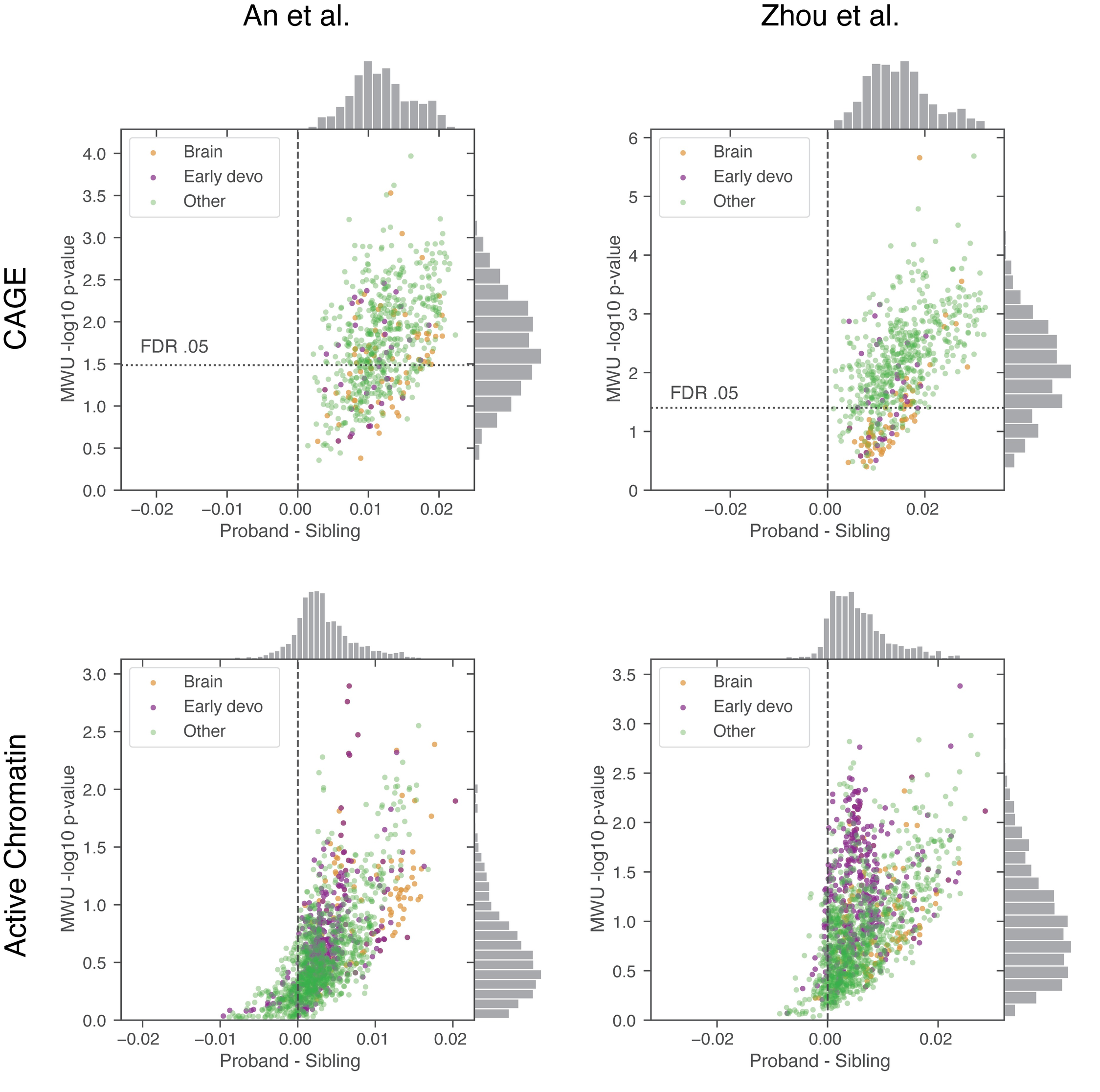

Supplement: S18 Fig — We predicted the influence of de novo variants found in proband and sibling genomes for human datasets. We studied two versions of the processed whole genome sequences by An et al. (left column) and Zhou et al. (right column) [43, 44]. We separated CAGE gene expression (top row) from active chromatin modifications DNase/ATAC/H3K4me3/H3K4me1/H3K27ac (bottom row). In each plot, points represent datasets, from which a predicted activity difference can be assigned to each variant. We focused on variants within 50 kb of a GENCODE mRNA TSS and scaled negative variant scores by 10 before taking the absolute value. On the x-axis, we plot the mean natural log score for all proband variants subtracted by the mean natural log score for all sibling variants. On the y-axis, we plot the log10 p-value from comparing proband to sibling scores using a Mann-Whitney U test. Many CAGE datasets show Benjamini-Hochberg q-values < 0.05; chromatin datasets demonstrate a similar trend toward greater scores for proband variants, but do not reach the same significance levels. (TIF) [file pcbi.1008050.s018.tif]
