# Supplementary material for: Cross-species regulatory sequence activity prediction
Source: PLoS Comput Biol. 2020 Jul 20;16(7):e1008050. doi: 10.1371/journal.pcbi.1008050 (PMC7392335; doi:10.1371/journal.pcbi.1008050)
Supplement: Attachment — Submitted filename: reviewer_response.pdf [file pcbi.1008050.s021.pdf]

Reviewer 1

Figure 5a appears to be reporting a major result though I am surprised the text is not highlighting the significance of what is being reported. In Fig. 5a the author is reporting case control differences with multiple of their mouse CAGE predictive models at a p-value better than 10^-6 with at least one model getting a p-value better than 10^-7. In contrast, in two recent high profile papers considering the same autism WGS data, in somewhat analogous analyses (Supplementary Fig. 4 from Zhou et al Nature Genetics, 2019 and Fig 1e from An et al, Science 2018) did not get p-values better than 10^-4 despite conducting an order of magnitude more statistical tests. Zhou et al Nature Genetics, 2019 also used a deep learning sequence based prediction strategy. There are a number of differences in how these analyses were conducted so it is hard from what is presented to know what is driving this large improvement in p-values. (I am assuming here the y-axis in Fig 5a is in base 10 scale though that was not explicitly noted)

I regret to inform the reviewer that I used natural log for the y-axis in (formerly) Figure 5a (now Figure 6a). I have changed that and other p-value plots to use log10 and labeled the axes accordingly. The minimum p-value achieved in the previous draft was 7.3e-4.

In our revised manuscript, I have revised and improved this analysis. As the reviewer suggested below, I downloaded Zhou et al.'s processing of the data, in which they made several different decisions that resulted in a smaller set of de novo variants. Guided by both data sets and the reviewer's suggestions, I redesigned the analysis and focus on robust observations. I now observe a minimum p-value of 5e-6, which is indeed lower than that achieved by An et al. or Zhou et al.

As the reviewer notes, these analyses differ from previous ones in several ways. An et al.'s analysis focuses on variant overlap with genomic segments with high signal in functional genomics assays. Machine learning models to predict sequence activity improve on such analyses, as studied by Zhou et al., by more precisely pinpointing variants that are most likely to influence regulatory activity. Analogous improvements have been observed for classification of disease variants in many prior papers.

Zhou et al. apply their DeepSEA method, which I demonstrate in Supplementary Figure 12 produces variant effect predictions with a weaker statistical relationship with GTEx gene expression eQTL statistics than Basenji. Furthermore, I observed the most significant p-values for CAGE datasets, which directly measure RNA abundance rather than less direct chromatin marks. Zhou et al. did not train models for CAGE or any other gene expression measurement. Furthermore, the authors synthesize their high-dimensional predictions for many datasets to a single score by fitting a classifier to predict Mendelian disease variants. Since current known Mendelian disease variants have no justifiable relationship with autism, I find this choice to be less than ideal. Instead, I compute Mann-Whitney U statistical tests for all datasets individually.

Altogether, these methodological distinctions amount to a different view on these data, no more valuable than the extensive analyses performed by An et al. and Zhou et al. I do not position this analysis to compete with theirs in any way; accordingly, it is not highlighted in the title or abstract of the paper. The objective of my manuscript is to demonstrate the value of training these machine learning models on both human and mouse genomes and demonstrate the value of variant predictions derived from mouse datasets. I hope the reviewer finds this analysis to be a compelling piece of data toward making that case.

Given that this has the potential to be a major result, I think the author should present more analyses and information allowing a reader to better understand the result, its relationship to previously reported results in the literature, and be convinced of the biological significance of the result as opposed to being driven by something technical. I have a number of comments/ suggestions in this regard:

i) Can the author explain why the p-values appear to be substantially more significant for the mouse CAGE data in Fig. 5a than for the human CAGE data in Sup. Fig. 8 even though the application of the model is within human? Maybe looking at the correlation of predictions for corresponding brain predictions between human and mouse and/or whether anything can be said about the specific variants better predicted using data from one species or the other would help with this.

Although the mouse CAGE data achieves lower minimum p-values relative to human CAGE data for the An et al. data, the order is reversed for the Zhou et al. data. In general, I agree with the reviewer that we would expect some loss during the transfer across species, so that the human data would be more informative. In a newly added analysis, I found that human-derived variant scores were more informative for classifying Mendelian disease and GWAS variants than mouse-derived scores, as one might expect. However, the difference was small. The similarity between human and mouse-derived predictions across the manuscript supports the value of this approach to transferring mouse regulatory activity models across species to human.

I examined variant proband variant scores for several compelling human and mouse datasets, but I could not detect any patterns that provided insight into the Mann-Whitney U test results. This exercise was challenging because these variant sets do not contain any true positives—most proband variants do not cause autism—so it is difficult to say which variants are “better predicted”. In general, any mouse dataset may produce better results in these analyses relative to human if it happens to have greater signal to noise or profile a highly relevant tissue or cell type, despite the cross-species transfer.

ii) Is the author currently including protein coding variants in the p-value calculations? If so what happens to the p-values if protein coding variants are excluded from the analysis? Protein-coding variants are known to already have a strong detectable association with ASD so it would be less interesting if the signal is coming from protein coding variants.

I thank the reviewer for raising this important point to clarify. Our methods have no awareness of protein coding sequence and no ability to identify variants that overlap and influence coding sequence. Variants in these data that happened to overlap coding sequence are evaluated purely for their influence on gene regulation. Thus, one would expect no difference in the results after removing variants that overlap coding sequence. I performed this analysis, removing 5% of variants, and the statistical enrichments different minimally, as expected. I describe this analysis in the main text and Supplementary Figure 14.

iii) An et al, Science 2018 corrected for parental age in computing p-values. If the author corrected for parental age what would happen to their reported p-values?

I follow Zhou et al. in computing Mann-Whitney U tests to compare allelic variant scores from probands versus siblings. In this framework, the number of mutations per individual (which covaries with parental age) does not affect the test results. Zhou et al. do no correct for parental age and verify that predicted mutation effects are not correlated with parental age. Altogether, I do not believe that correcting for parental age is necessary, nor am I certain how one might go about doing it. If the reviewer could clarify their concern, I would be happy to consider it.

iv) Zhou et al, Nature Genetics 2019 used a different set of de novo mutational calls from An et al, Science 2018 and used here, with a notable difference of excluding variant calls overlapping repeat elements. If the author applies their predictions on the same set of de novo mutation calls from Zhou et al what p-values result?

As described above, I now conduct an analysis on variant sets from An et al. and Zhou et al. and focus on robust observations. In general, Zhou et al. enrichments tend to be less significant, likely because of having about 1/2 the variants called in the An et al. sets. All results are described in Supplementary Figures 13 and 15 and Supplementary Table 2.

v) Are these strong p-values unique to CAGE data or can they also be seen in chromatin data?

In my revised analysis of de novo variants in autism, I studied both CAGE and active chromatin data sets (DNase, ATAC, H3K4me3, H3K4me1, H3K27ac). CAGE enrichments are stronger than the chromatin data. This is likely attributable to its focus on RNA gene product, which is more directly relevant to disease influence. I describe this analysis in the manuscript and Supplementary Figures 13 and 15 and Supplementary Table 2.

vi) To what extent can the author's predictions recall case de novo mutations at high precision? This type of evaluation could be more relevant than a p-value across the full distribution since the expectation with de novo mutations is that their contribution is through a smaller number of larger effect variants

In place of Mann-Whitney U tests, I computed AUROC statistics on the task of classifying proband versus sibling variants. The most significant enrichments lead to AUROC 0.508, suggesting that these data produce classifiers that are better than random (as supported by the statistical testing perspective), but probably not useful in that capacity. As the reviewer suggests, and supported by the fact that both children have nearly the same number of variants, the large majority of variants seem to be unrelated to autism. Training classifiers depends on a certain level of signal to noise that is not available in these data at the variant level.

However, classification at the level of individuals (rather than variants) is more tractable. In Figure 6c, we demonstrate that our variant scores deliver value towards training such classifiers. A maximally useful version of such a classifier would benefit from additional sources of data such as coding mutations and conservation statistics and is outside of the scope of this work. However, I hope these variant scores will be useful for groups interested in such work.

vii) The methods states “We filtered these variants for SNPs”. Can the author provide more details of what SNP list was used for filtering, how many variants were filtered, and whether this is causing difference with previous analyses?

By this statement, I meant that I removed insertions and deletions from the variant sets. I have clarified this point in the text.

viii) The author should provide as a supplementary data or a file on a website that provides their scores for each de novo mutation based on each CAGE dataset

I have made the variant scores for both An et al. and Zhou et al. available from https://console.cloud.google.com/storage/browser/basenji_barnyard/sad/autism/ and added the link to manuscript and github section dedicated to the manuscript.

Another major comment I have about this manuscript is that I think the author should evaluate the extent to which improvements for human predictions when training with mouse is occurring because of the diversity of sequence space the mouse provides as motivated in the introduction, or if a similar or better improvement would be seen by using an equivalent number of additional datasets from human. The author only used ENCODE and FANTOM5 data in human so is far from exhausting the available data sets on regulatory activity collected directly in human as there are large datasets from other consortium (e.g. Roadmap Epigenomics, Blueprint) and databases that provide uniform reprocessing of major data repositories (e.g. ChIP-atlas and ReMap). The framework the author is proposing might actually make more sense when the primary interest is in a mammal with far less functional genomics data available than mouse or human.

The reviewer makes an excellent point that I had not fully demonstrated that the models improve because of more sequences and annotations, rather than just more annotations. To explore this, I split all human datasets into eight folds. For each fold, I held out those datasets and trained a model only on the other seven folds. For each dataset, I averaged test set accuracy for the seven folds that did train on it, each of which had a different 12.5% of the datasets held out. If it were true that adding more annotations benefited model training and accuracy, then these models would suffer from the held out targets and show reduced accuracy relative to the full model. Instead, these models achieved greater accuracy than the full data model for 51.0% of the datasets. The average PearsonR across targets was greater for the held out training runs by a minuscule 0.0015. Thus, more annotations alone do not increase model accuracy, but additional sequences and annotations do. I describe these analyses in the main text and Supplementary Figure 6.

Additional comments
*There are similarities between what the author is doing here and Chen et al, PloS Comp 2018. The authors cite that manuscript in the results section, but I think the author should acknowledge this prior work and clarify the contribution of this manuscript to prior work up front in the introduction.

I have rephrased the introduction and other points in the manuscript to more clearly highlight this prior work.

* On page 10, the authors say their procedure can suggest high level annotations for 6/9 of the unknown single cell clusters. Is this leading to different/better suggestions than using nearby gene expression of cluster peaks?

In their manuscript introducing the single cell ATAC-seq atlas for mouse, Cusanovich et al. attempt to annotate clusters using just such an approach, in which they impute gene expression from ATAC-seq signal near genes, and consider known marker genes. These nine unknown clusters could not be unambiguously annotated by this approach. We have clarified this point in the text.

* The methods could be more detailed/precise in places. For example, how was the threshold t_c chosen? Another example, it is stated "We found that training several epochs on only one genome or the other after the full joint procedure improved validation and test set accuracy." How many epochs did the author actually use for this final step and how was it determined?

t_c specifies the soft clipping threshold for each dataset. I manually chose t_c per experiment and source by inspecting the maximum values, aiming to reduce the contribution of rare very large values that one would not expect to generalize to other genomic locations. Via this procedure, I set t_c=384 for CAGE with its high dynamic range, t_c=32 for ENCODE with its far lower range, and t_c=64 for GEO.

I have clarified the fine-tuning stage in the methods, which is now described as follows. "We found that training on only one genome or the other after the full joint procedure improved validation set accuracy. We evaluated the model on the validation set after every epoch and stopped training after 10 epochs without improvement, returning to the previous model that achieved the minimum validation loss. The model studied here was fine-tuned for 8 epochs on the human data and 20 epochs on the mouse data."

* It is stated "Stricter parameter settings created a single large connected component that did not allow for setting aside enough validation and test sequences". This does not directly address whether the current settings are adequate to avoid the memorization issue

Careful splitting of sequences across species into train/valid/test sets addresses the concern that test set accuracy may be inflated for the jointly trained model. Although it is true that a small amount of orthologous sequence remains even after our procedure, there are several compelling pieces of evidence to suggest that this is not problematic.

First, the proportion of the human and mouse validation and test set nucleotides that have orthologous sequence across genomes in the training set is always <1%, making it unlikely to contribute meaningfully.

Second, the regulatory sequence turnover between these two genomes is extensive and even ostensibly conserved promoters have substantial differences between them (Vierstra 2014). I would be more concerned if the two genomes were human and chimpanzee, for which orthologous sequences are far more similar.

Third, model comparisons in genetic variant space are unaffected by potential orthologous train/test leakage because we compare one allele to the other. If a model has memorized sequences, rather than learned general principles of gene regulation, then it will not be able to make accurate and meaningful predictions for variant effects. Our model predictions for human genetic variants have a strong statistical relationship with GTEx summary statistics via signed LD profile regression and disease variants, indicating that they have learned general principles of gene regulation. Furthermore, in every variant analysis task, the jointly trained model outperforms the model trained on human alone.

Altogether, we believe that the train/valid/test splitting procedure effectively reduced leakage and models trained jointly on both genomes are more accurate and useful.

Vierstra, J., et al. (2014). Mouse regulatory DNA landscapes reveal global principles of cis-regulatory evolution. Science 346(6212), 1007 1012. https://dx.doi.org/10.1126/science.1246426

* page 12 line 239, “negative predictions” used before explaining “negative predictions” at the bottom of the page

I now define this term at its first mention.

*there are ? in sup. Fig 1 and 2 legends

I double checked that all citations and references in the supplementary legends of the revised manuscript are properly formatted.

Author should provide prediction values for autism mutations part of Fig 5a analysis

I have made the variant scores for both An et al. and Zhou et al. available from https://console.cloud.google.com/storage/browser/basenji_barnyard/sad/autism/ and added the link to manuscript and github section dedicated to the manuscript.

Reviewer 2

1. In terms of methodology, it lacks novelty. The strategy is simply concatenating two datasets from different organisms and making sure that homologous sequences appear in the train/test set together. This is hardly a new computational development effort. The author also said that a novel transfer learning approach was developed, but it seems that this is just training the model on mouse dataset and predicting on the human dataset, which is not exactly transfer learning. There are no methods such as domain adaption developed for the specific problem. This is more like a cross-species validation/assessment rather than a transfer learning approach.

Although other research groups have explored training machine learning models on data from multiple species, my effort here is unprecedented in scope and final product. That is, training large state of the art deep convolutional neural networks on thousands of human and mouse datasets, including gene expression profiles, has not been done before. My experiments demonstrate that this strategy is effective and produces models that predict noncoding genetic variant effects with greater statistical concordance with GTEx than alternative available approaches. I have released these noncoding variant predictions and the models along with this manuscript to enable further research into the genetic basis of disease and they are being actively used (e.g. Dey et al. 2019).

I appreciate the reviewer's point that I have used the term "transfer learning" imprecisely. I have removed all mentions of "transfer learning" and replaced them with more specific descriptions.

Dey, K., Geijn, B., Kim, S., Hormozdiari, F., Kelley, D., Price, A. (2019). Evaluating the informativeness of deep learning annotations for human complex diseases. bioRxiv https://dx.doi.org/10.1101/784439

2. In the first section of the results, the author compared the performance of a jointly trained model versus the model trained with the independent dataset. However, there are existing methods for cross-species/cross-cell type functional genomic signal prediction. For example, Chen et al. PLOS Computational Biology 2018 (PMID: 30286077) and also Lan et al. Int J Mol Sci. 2019 (PMID: 31336830). Although the setting is not exactly the same because one is binary classification while this one is regression, it is rather straightforward to adapt those methods to the setting in this work. The comparison with existing methods is lacking.

Chen et al. "hypothesized that enhancers active in different mammals would exhibit conserved sequence patterns" and "evaluated the extent to which sequence patterns that are predictive of enhancers in one species are predictive of enhancers in other mammalian species". Chen et al. do not explore training on multiple genomes, and they do not study human genetic variants. Their analysis is similar to my section "Regulatory sequence activity models transfer across species". The purpose of this analysis in my manuscript is to serve as a logical stepping stone to studying human genetic variant predictions derived from models trained on mouse data. I reference the contributions of Chen et al. in the introduction and that section.

Lan et al. propose a method for learning models for TF binding that predict accurately in new and different cell types for which one does not have training data. My manuscript does not address this question. Lan et al. do not study from multiple species. However, the techniques that Lan et al. use

for their cross-cell-type task may be applicable, and I have added a reference to their manuscript in discussing the potential of more sophisticated versions as future work.

The reviewer notes that these models both predict binary peak classifications as opposed to continuous signal regressions. We benchmarked these two alternative approaches in a controlled setting in previous work, and regression produced more accurate models (Kelley et al. 2018). Furthermore, regression is required for gene expression measurements with a large dynamic signal range.

Adapting either of these authors' models would not be straightforward. Given that neither group shared my research objective in this work, I do not believe a comparison would be insightful. In contrast, the DeepSEA method of Zhou et al. shares my objective of producing noncoding variant effect scores that predict gene expression. Thus, I added a comparison of variant effect predictions from DeepSEA and Basenji trained in various modes, benchmarked with GTEx summary statistics. Basenji scores have a stronger signed relationship with these gene expression measurements than DeepSEA.

Kelley, D., Reshef, Y., Bileschi, M., Belanger, D., McLean, C., Snoek, J. (2018). Sequential regulatory activity prediction across chromosomes with convolutional neural networks. Genome research. 28(5), 739 750. https://dx.doi.org/10.1101/gr.227819.117

Zhou, J., Troyanskaya, O. (2015). Predicting effects of noncoding variants with deep learning–based sequence model. Nature Methods 12(10), 931-934. https://dx.doi.org/10.1038/nmeth.3547

3. For the third subsection in Result. The author showed the importance of the orthogonal information brought in by the mouse scATAC signal by including the principal components of human signals in the background model. The results showed that the mouse signals can be important, but the level of importance is still not clear. It would be interesting to design the experiment when the human signal and mouse scATAC signal are jointly tested. This would help to quantitatively analyze the importance of this orthogonal information by comparing the Z-score from the human signal and mouse signal. If in this test the mouse signal consistently shows up at the top, then it can demonstrate the usefulness of this method to generalize signals from another organism to the human better. I think that this needs to be addressed.

I agree with the reviewer that a symmetric joint analysis of the human and mouse data would be interesting. Unfortunately, SLDP cannot perform such an analysis; every annotation provided is analyzed independently, conditional on the background model. Comparing signed annotations to GWAS summary statistics is very complicated due to linkage disequilibrium, and SLDP represents the current state of the art. Extending to joint analyses is beyond the scope of this work. I have personally communicated this objective to the primary authors of that work.

In addition, these annotations are frequently very correlated with each other. Partitioning saliency to correlated features in any statistical machine learning exercise is fragile and challenging to interpret. The conditional analysis enabled by SLDP establishes specific cases where the mouse data delivers novel value beyond human data with statistical significance. In newly added sections and Figure 5, we also now benchmark disease variant classifiers that make use of features derived from human and mouse datasets. In the case of Mendelian and GWAS variants, mouse-derived features

boost classifier performance with statistical significance. Interestingly, models trained on mouse-derived features alone nearly match the performance of human-derived features. Thus, multiple lines of evidence point to a contribution of these data.

Minor points:
Figure 1,3,4 are barely readable. The font size is really small and thin.

I share the reviewer's concern that printed versions of these figures can be difficult to read. I have increased the font sizes for all primary figures, and I will work with the editors to ensure that final versions are legible.

Reviewer 3

The authors demonstrate that CAGE eRNA profile prediction improves more than prediction of other functional genomics features from ENCODE/Roadmap. Are there substantial differences between functional genomics profiles or cell types of origin in terms of the improvement provided by the method introduced here?

I thank the reviewer for encouraging this deeper examination of how performance differs by functional genomics experiment. I have added a Supplementary Figure 3, splitting the Figure 2 accuracy scatter plots by experiment type for the most frequent 24 experiments. While I would not say that there are substantial differences, some dataset classes fare better than others. In particular, H3K9me3 predictions are slightly worse after joint training on human and mouse.

Splitting accuracy comparisons by cell type would be too fine a resolution, as most cell types only have one or a few datasets. However, Meuleman et al. 2019 considerable work into annotating all ENCODE and Roadmap DNase-seq datasets, including a “System” annotation that merges datasets into 15 categories such as “Renal”, “Hematopoietic”, and “Musculoskeletal”. I plotted test set accuracy for models trained on human alone and human/mouse jointly for each category in a new Supplementary Figure 4. Pearson R improves by an average of 0.006 across all of these DNase datasets. Within the various categories the improvement differed slightly. “Musculoskeletal” datasets improved by 0.008, which was significantly greater than the remainder by Mann-Whitney U test with p-value 2e-7. In contrast, “Connective” datasets improved by 0.004, which was significantly less than the remainder with p-value 1e-10. I was unable to discern a pattern that provided insight into why some categories improve more or less than others with joint training.

Meuleman, W., Muratov, A., Rynes, E., Halow, J., Lee, K., Bates, D., Diegel, M., Dunn, D., Neri, F., Teodosiadis, A., Reynolds, A., Haugen, E., Nelson, J., Johnson, A., Frerker, M., Buckley, M., Sandstrom, R., Vierstra, J., Kaul, R., Stamatoyannopoulos, J. (2019). Index and biological spectrum of accessible DNA elements in the human genome. bioRxiv. https://dx.doi.org/10.1101/822510.

Line 102–108. To me “regulatory programs” implies the particular combinatorial patterns of many regulatory elements that drive specific patterns of gene expression. Thus, in my view, it is not necessarily true that the regulatory programs themselves are conserved, but rather that the components of these programs are conserved. To extend the “program” analogy further: human and mouse may have different regulatory programs, but these programs are written in the same language.

I appreciate the reviewer perspective on how this term is perceived by others in the field. I have changed all occurrences of “regulatory program” in the manuscript, often substituting the term “regulatory grammar”, in line with the reviewer’s suggestion of a similar “language”.

In the last Results section “Mouse-trained models highlight mutations relevant to human neurodevelopmental disease”:

- The predictive power of summing the negative predictions in the neonate cerebellum dataset at predicting cases from controls (Figure 5C) seems quite poor. Is it possible to benchmark this performance with the deep learning model used in Zhou et al. Nature Genetics 2019 (PMID: 31133750)?

Some children develop autism spectrum disorder, adjacent to highly genetically related parents and siblings who present as neurotypical. What causes this to happen looms as an enormous question in the field of biomedical research. Although many gene mutations have been discovered that are statistically enriched in autism patients, these do not amount to a compelling explanation of why some kids get autism and others do not. Nobody in the field has such a classifier, and its introduction would represent a monumental achievement with tremendous and far-reaching implications.

With that context, I agree that the predictive power that I demonstrate for classifying autism probands versus their siblings is quite poor. Nevertheless, I have made use of a single annotation (i.e. no gene annotations or conservation) and achieved a classifier that is significantly better than random. I performed this analysis to demonstrate that there is value in our suggested multi-genome training procedure and cross-species variant effect predictions, and I believe that it does.

Zhou et al. studied a single summarized variant score annotating de novo variants in these autism simplex families. This single summarized score is derived from fitting a regularized linear model on predictions from 2002 epigenomic datasets to classify a curated set of regulatory mutations from HGMD. Although they detect that proband variants have statistically significantly greater scores than sibling variants, they did not attempt to use these scores to classify probands from their siblings.

Given that it was not the intention of my work or Zhou et al. to develop an autism individual classifier, I do not believe a fair comparison between our two methods could be performed. In this revision, I have added a comparison to Zhou et al.'s DeepSEA method on GTEx eQTL data in Supplementary Figure 12 that I believe is fair and compelling.

- In the cross-specifies prediction, such as Figure S5d, the mouse model's prediction is worse than human predictions. Have you analyzed the sequences that the mouse model failed to predict? Could it be partially driven by the very different GC content landscape in the mouse genome compared to the human genome?

My objective in that analysis was to demonstrate that mouse-derived predictions for human sequences contain meaningful information about the measured human regulatory activity. There are many reasons to expect that the mouse predictions would not match the concordance of the human predictions. First, you will always observe that a model trained on the same experiment will achieve the best accuracy on held out sequences for that experiment (Kelley 2018). I.e. if you train models on two human biological replicates A and B. Then the model trained on A will achieve better accuracy predicting measurements from A on held out sequences and the model trained on B will achieve better accuracy predicting measurements from B on held out sequences. Thus, even if I had performed the analysis with separate human biological replicates, we would inevitably observe a similar trend.

Nevertheless, how regulatory grammars differ between genomes is an interesting topic, and I am happy to comment further on it in this analysis. I plotted GC% in a 1,000 bp window versus the residual difference between the observed and predicted signal for predictions derived from the human versus mouse data as Supplementary Figure 9. Indeed, there are some differences present that may account for some of the worse accuracy of the mouse predictions versus human data. For all three tissues, the correlation has greater magnitude for mouse versus human. For example, in the cerebellum, the human residuals have a -0.06 correlation with GC%, but the mouse residuals have a 0.11 correlation. Thus, GC% calibration or more sophisticated forms of domain adaptation may be an interesting avenue to further improve cross-species transfer. In addition to the supplementary figure, I now describe this observation in the main text.

Kelley, D., Reshef, Y., Bileschi, M., Belanger, D., McLean, C., Snoek, J. (2018). Sequential regulatory activity prediction across chromosomes with convolutional neural networks. Genome research 28(5), 739 750. https://dx.doi.org/10.1101/gr.227819.117.

- The result section emphasizes that the mouse-trained model can be applied to understand human disease. While this point is true, given the abundance of human data and potentially better performance of human model (Figure S8) in this autism cohort, it would be useful to also demonstrate that incorporation of mouse data with the human data helps. More specifically, demonstrate that the human predictions from the human-mouse joint model is better than the human predictions from the human only model.

The reviewer makes a compelling argument that my claims of superior variant effect predictions from joint genome training would benefit from more evidence. The manuscript now includes several diverse sources of such evidence, which all support the claim that training on multiple genomes benefits variant analysis.

First, I compute the statistical relationship between variant effect predictions for human CAGE datasets to GTEx gene expression eQTL statistics using signed LD profile regression (SLDP). Predictions from the jointly trained model have larger Z-scores relative to predictions from the model trained only on human, described in Supplementary Figure 11.

Second, I performed conditional SLDP analysis to assess whether variant predictions for mouse datasets deliver novel value above and beyond the information in human dataset predictions. Indeed, there were several examples that met this high bar, described in Supplementary Figure 10.

Third, I collected variants implicated in Mendelian disease and GWAS via fine-mapping and trained classifiers to distinguish these variants from carefully controlled negative sets. In both cases, classifiers trained on variant predictions for human datasets from the jointly trained model outperformed classifiers trained on predictions from the single genome model. Furthermore, classifiers trained on variant predictions for both human and mouse datasets outperformed classifiers trained on only human dataset predictions. These analyses are described in newly added Results sections and Figure 5.

Altogether, I believe the case is now very strong for the superiority of this training approach for studying the regulatory activity of human genetic variants.

- As a final general comment, the impact of the manuscript would be stronger if (especially in the applied sections of the Results) the authors could compare to other state-of-the-art machine-learning-based methods for regulatory variant interpretation.

To address the reviewer's concern, I added a comparison of Basenji-derived variant effect predictions to an alternative deep learning approach DeepSEA. In Zhou et al. 2019, the DeepSEA authors introduced their latest version of this model trained to predict 2002 epigenomic peak sets. Using this model, I computed variant effect predictions for all 1000 Genomes variants. The epigenomic datasets studied by Zhou et al. are incompletely annotated with their ENCODE or Epigenomics Roadmap identifiers. I manually aligned these data to the datasets studied in this manuscript by searching the ENCODE site to identify 100 unambiguous matches for DNaseI-seq experiments. Finally, I computed signed LD profile regressions (SLDP) for these variant scores versus all GTEx tissues. Basenji predictions resulted in significantly greater SLDP z-scores, indicating greater concordance between signed accessibility predictions and gene expression, for 70% of these datasets. I have added this analysis to the manuscript as Supplementary Figure 12.

Minor comments:
The Introduction (Lines 25 – 32) does not fully cover the previous literature on the prediction of genomic data using multiple species. in paragraph. The author did mention some previous work in the Results section (Line 114). It would be better to cover this background more fully in the Introduction and to cite other relevant work, such as Cohn et al. 2018 (https://www.biorxiv.org/content/10.1101/264200v2).

I thank the reviewer for suggesting this reference. I have rephrased our introduction to highlight this prior work and now cite Cohn et al. We note that Cohn et al. do not describe careful splitting of train/valid/test sequences that consider homology between genomes. Thus, their results are challenging to interpret and do not conclusively demonstrate that multi-genome training leads to improved generalization accuracy.

Line 20. The author mentioned that "Individual human genomes differ only slightly from each other, so acquiring functional profiles for more humans is unlikely to provide this boost."
I don't necessarily agree with the point. Although small percentage of the genome differs between individuals, the total amount genetic variants is large and gives rise to the diversity of human phenotypic traits and diseases. I would acknowledge that multiple different ways of obtain more training data may be helpful.

I appreciate the reviewer's optimism on this potential source of new training data. I have rephrased the sentence to remove my pessimism and more plainly state the facts.

Line 191 – 210. In Figure S6, is the mouse single cell ATAC-seq dataset used in the SLDP? I didn't see any of the mouse scATAC data sets among the significant ones.

In the analysis for Supplementary Figure 10, I studied the CAGE and DNase/ATAC datasets separately. Panels (a) and (b) display results from the CAGE analysis and do not contain the UW single cell ATAC data. Panel (c) displays results from the DNase/ATAC analysis, which does include the UW single cell profiles. The second most enriched dataset for GTEx liver is the UW hepatocyte

profile. Several single cell clusters representing proximal tubule also emerge. In the figure legend, I point the reader to the mouse single cell hepatocyte result. I have revised the legend to be more clear.

Figure 3c,e: It would be helpful to use a simpler color map with white at 0 and a gradient to a single color to represent positive values and to a different single color for negative values. See: https://www.oreilly.com/library/view/fundamentals-of-data/9781492031079/ch04.html

I substituted a different colormap with white at 0 for Figure 3 panels c and e.

Line 297–299. This claim is not accurate. This manuscript is not the first to propose that machine learning models can be used to extract relevant features independent of alignability to apply across species. In other words, previous approaches for applying machine learning methods to infer regulatory activity and functional genomics feature profiles across species do not all rely on alignability of the underlying sequences.

I have removed this claim. However, my literature search did not uncover any prior methods that fit this criteria. I would very interested to know which references the reviewer is thinking of.
