# Supplementary material for: Cross-species regulatory sequence activity prediction
Source: PLoS Comput Biol. 2020 Jul 20;16(7):e1008050. doi: 10.1371/journal.pcbi.1008050 (PMC7392335; doi:10.1371/journal.pcbi.1008050)
Supplement: Attachment — Submitted filename: reviewer_response.pdf [file pcbi.1008050.s022.pdf]

Reviewer 1

1. I previously raised the point about whether the increased predictive power was due to unique information in mouse sequence data, or whether comparable predictive performance increase could be obtained from incorporating additional human data. I pointed out that there are many additional sources of human data not being used. In response, the author showed there was basically no impact on predictive performances when downsampling the current set of human data to 7/8 the size.

I did not find this analysis fully satisfying for two reasons. One reason is that holding 1/8 of the human data sets out amounts to 664 human data sets, while there was 1,643 mouse data sets used, thus the comparison of value of additional human vs. mouse data is confounded by the number of data sets. The other reason is that the analysis is effectively assuming the current human data being considered is representative of all available human data, which is likely not the case. I expect that when one gets data from an additional source, even if it is from the same species, it will contain more new information compared to data obtained in the same way as the data being currently considered.

The reviewer raises the concern that holding out 664 human datasets is not sufficient to observe a a possible decrease in generalization accuracy, given that the mouse data adds 1,643. The reviewer previously raised the concern that the improved generalization accuracy for human datasets obtained by adding mouse datasets to the multi-task learning problem could be attributable to the addition of any new datasets, including more from human, rather than specifically ones from a unique genome. Answering the following question would rule out this possibility—given that I have 5,313 human datasets, is there any marginal benefit to a new dataset? By holding out 664 datasets (1/8), I have demonstrated that there is no marginal benefit to 664 new datasets, given 4,649 datasets (7/8).

The reviewer now raises the concern that holding out 1/8 may not be sufficient in magnitude to observe this marginal benefit. To address this concern, I repeated the hold out experiment with 1/4, 1/3, and 1/2 of the datasets. The latter two experiments both hold out more datasets than the 1,643 added from mouse. In each of these experiments, human generalization accuracy is stable. I revised Supplementary Figure 6 to include these new results. Thus, the multi-task learning benefit of novel human datasets has saturated.

The reviewer raises an additional concern that these human datasets may not be representative of all available human data and that human data from additional sources would contain new information. I believe this is a very unlikely scenario for the following reasons:

(1) The human datasets are derived from varied sources (ENCODE, Epigenomics Roadmap, and FANTOM consortiums) that are standard in the field due to their having been generated and analyzed using carefully vetted experimental protocols and bioinformatic pipelines. They represent an enormous variety of tissues and cell lines.

(2) The mouse datasets are primarily derived from these same sources—1,109 from ENCODE and 357 from FANTOM). The data were generated by the same experimental protocols and bioinformatic pipelines. Thus, any possible benefit due to novel sources of data would be minimal.

(3) The experiment described above in which 1/2 of the data is held out introduces significant data source variation, as many tissues and cell lines will be entirely excluded from training.

Finally, I want to convey that acquiring, processing, and quality control checking data for this type of machine learning analysis is a substantial task that has required years of personal work. Acquiring and preparing >1,643 novel human datasets that are sufficiently different from the existing ones, followed by training and quality control analyzing models on the new data would require many months of work. I believe that such work is not a worthy investment for the reasons outlined above.

2. The procedure for the autism analysis was changed to remove variants not within 50kb of a TSS, and to weigh negative predictions 10 times more than positive predictions. I did not suggest either of those specific changes. While the p-values improved (when everything is compared in log10 space), it is now more difficult to interpret the p-values. The reason is that I assume there was some type of multiple testing going on in selecting these parameters, but that procedure was not described and any additional tests were not reflected in a multiple testing correction, but should be. Also, I think it would be informative to report as was done in the original submission how well the scores does directly without going through this filtering and reweighting. The reported predictive performance at the individual level for the top variant feature might also be inflated by multiple testing issues.

I thank the reviewer for highlighting remaining issues in this analysis. I chose these procedural changes after carefully reviewing the procedures of An et al. and Zhou et al. and studying datasets from both previous papers.

As the reviewer points out, the procedure has two hyperparameters—a variant distance to gene TSS threshold and a scaling factor for the negative predictions. I did not use a TSS threshold in the initial analysis, but learned of the value of such a filter from its application in the Zhou et al. analysis. Because the sequence-based machine learning model is gene agnostic, this filter is sensible to highlight mutations that are more likely to influence gene expression, in contrast to mutations in gene deserts. These distant mutations will still often have nonzero CAGE predictions due to the assay's sensitivity to enhancer RNAs.

The second hyperparameter to scale the negative predictions is required for an effective analysis because negative predictions are clearly more important in these data than positive. Zhou et al. did not require an explicit analogous hyperparameter because they study predictions output by a random forest classifier that will implicitly learn this property of the variant scores.

I acknowledge the challenge that the reviewer highlights in evaluating p-values from analyses that require hyperparameters. To assuage concerns that the results are sensitive to these hyperparameter choices, I have added two additional supplementary figures that display results of parameter sweeps for variant distance to TSS (Supplementary Figure 13) and negative predictions weight (Supplementary Figure 14). Statistical test results are robust to these parameter choices around similar values to those chosen. Under this new procedure, the An et al. statistical tests do not produce an FDR significant q-value less than 0.1 without a gene filter <500kb (which includes 90% of variants). I now note this dependency in the main text.

I also want to emphasize that the objective of this section of the manuscript is to evaluate the hypothesis "that predictions using models trained on mouse data would also distinguish [autism] and perhaps provide additional insight via novel developmental profiles". I accept the

commendable prior work performed by An et al. and Zhou et al., and do not argue that I have achieved better results. I do not intend to position this analysis as a contribution to the autism research field, and I have not highlighted it in the title, abstract, introduction, or discussion which are dedicated to demonstrating and evaluating multi-genome training for regulatory sequence activity prediction models. Instead, I aim to explore whether previous results can be reproduced through the lens of the mouse data and report my experience. Accordingly, I have removed all text highlighting the magnitude of the p-values and explicitly do not compare the p-value magnitudes to prior work with these data. I have also added a statement to the text to caution readers, "P-value magnitudes should be interpreted cautiously given the challenge of multiple hypothesis correction in an exploratory analysis with hyperparameters."

3. Related to the previous point, in the original submission there was emphasis on brain being the important cell/tissue type, but now the emphasis is on earlier development stages and the brain does not standout. It is concerning the biological conclusions are that sensitive to these specific parameter choices. Further raising concern, the text mentions 'whole body embryo E16' being the leading dataset, but according to Supplementary Table 2, 'whole body embryo E16' is third most significant and 'urinary bladder, adult' is actually the most significant.

I share the reviewer's concern that some observations changed between the first and final version of this analysis. Suggestions that the reviewer made during the first round of reviewer were extremely helpful to focus on robust observations from the strongest possible analysis. In particular, studying the Zhou et al. processing of this data alongside the An et al. processing guided me away from initial analysis choices that were not optimal. I believe this represents the review process working at its best.

In the current version, I have studied this data comprehensively, including from the following angles:
(1) Two separate processing pipelines of the whole genome sequencing data.
(2) CAGE and active chromatin datasets.
(3) Mouse and human datasets.
(4) With and without a coding gene filter.
(5) Parameter sweep over variant distance to gene TSS filters.
(6) Parameter sweep over negative prediction scale factors.
Thus, I believe that the current report represents accurate and robust observations. As I described in my response above, I intended to evaluate the hypothesis "that predictions using models trained on mouse data would also distinguish [autism] and perhaps provide additional insight via novel developmental profiles". I believe this thorough analysis accomplishes that objective.

I have changed the language around the whole body embryo E16 dataset to correctly describe its status relative to other datasets as "a leading developmental dataset".

4. The author asked for clarification on the point about parental age. An et al described in their paper their procedure for controlling for parental age and provide parental age annotations with it. Zhou et al did not correct for parental age, but did show that parental age was not correlated with their score. While the author's score might not be correlated with age, that was not shown. Zhou et al not seeing a correlation with parental age for their score, does not imply that the authors' scores is not correlated with parental age, though does make that possibility more likely.

I have added Supplementary Figure 15, which verifies using regression analysis similarly to Zhou et al., that there is not evidence that the variant effect predictions depend on the mother or father's age at birth.

5. On the point about annotating unknown clusters from Cusanovich et al. I don't think its been shown that the author's procedure is actually leading to better annotations opposed to being willing to annotate a cluster when there is still more uncertainty. For example for cluster 5.6, a number of different brain regions rank highly in Fig. 4b of the author's manuscript, but from the tissue type annotations of the cluster in Fig 2d of Cusanovich et al, it was already clear this cluster was related to the brain.

I thank the reviewer for their feedback that this analysis is not sufficiently compelling; I have removed the suggestion that unknown clusters can be labeled by this procedure.

6. I felt the added comparison with EIGEN and FunSeq2 is confounding two different questions. One question is whether the features produced by Basenji add value to variant prediction over features considered by other variant prioritization methods. The other question is whether there is an advantage to integrating a set of features in task-specific ways that is optimized for the evaluation of the task. Only the Basenji features were integrated in task-specific ways, but it is possible integrating the features of EIGEN or FunSeq2 in a way that is optimized for the evaluation would have led to even better performance than what is being reported for Basenji features.

I agree with the reviewer that these two questions are confounded in thus analysis. I did not intend to answer the second question regarding "integrating a set of features in task-specific ways that is optimized for the evaluation of the task". Therefore, I have removed EIGEN and FunSeq2 from the figures and text.

Reviewer 2

The author addressed my major concerns on the performance evaluation by including an extra section of the comparison with DeepSEA, another state-of-the-art machine learning-based model for regulatory variant interpretation, which demonstrates the advantage of this approach. The author elaborated on the differences of this work's object with the goal of the existing work and released the noncoding variant prediction results which clarify the contribution of this work. The author did improve the quality of the figures to a certain extend. However, I still think the font size of Figure 1,3,4 is too small. The authors and the editors should work together to ensure the quality of the figure.

I have further increased the smaller font sizes in Figures 1, 3, and 4 and commit to working with the editor to ensure their quality in a final version.
