# Supplementary material for: Cross-species regulatory sequence activity prediction
Source: PLoS Comput Biol. 2020 Jul 20;16(7):e1008050. doi: 10.1371/journal.pcbi.1008050 (PMC7392335; doi:10.1371/journal.pcbi.1008050)
Supplement: Attachment — Submitted filename: reviewer_response.pdf [file pcbi.1008050.s023.pdf]

Reviewer 1

Unless I am misunderstanding something, I believe the author is making a basic error that is causing in part an unsupported biological conclusion to be reached in the Autism analysis.

I previously raised concern that early development was being emphasized as being relatively important, while under a different setting of the parameters it wasn't. Additionally I was concerned that the emphasis was being placed on early development when 'urinary bladder, adult' was the most significant hit for the An et al Mouse data sets.

The author in the text and response is saying the conclusions were robust to two different processings of the data. However, despite what the author is saying in the text and the response, for the Zhou et al data, based on what is in Supplementary Table 2 the p-values actually appear less significant for embryo/neonate CAGE data than other CAGE data.

I computed the median p-values for 218 embryo/neonate data CAGE data sets and 139 non-embryo/neonate CAGE data sets (note the text said it was 219 vs. 138 so there is a discrepancy of one) and obtained the following:

Median Zhou et al embryo/neonate: 0.020
Median Zhou et al not embryo/neonate: 0.009
Median An et al embryo/neonate: 0.002
Median An et al not embryo/neonate: 0.006

Additionally, even for An et al data I don't think it is accurate to describe embryo/neonate datasets as being 'prominent among these associations' as I am not seeing them in the top part of the rankings of the most significant CAGE data.

I gravely apologize for an error in presentation that led the reviewer to conduct this very thorough and much appreciated review of the supplementary table statistics. The reviewer is correct that the 218 mouse embryo/neonate CAGE datasets do not have significantly lower p-values in the Zhou et al. processing of the data. The only consistent trend that I observed across the An and Zhou datasets was for 13 *whole body* embryonic and neonate developmental stages. In the text describing the An et al. analysis, I mistakenly referred to a larger set of embryonic and neonate profiles that includes myriad specific tissues. These do have significantly lesser p-values in the An et al. data (p-value 6.4e-4), but they do not in Zhou et al.

I have reproduced the reviewer's table with the new sets:
Median Zhou et al whole body embryo/neonate: 0.0038 (p-value 4.5e-3)
Median Zhou et al not whole body embryo/neonate: 0.0141
Median An et al whole body embryo/neonate: 0.0005 (p-value 7.7e-4)
Median An et al not whole body embryo/neonate: 0.0032

I have corrected the sentence in the manuscript to describe the whole body datasets. The reviewer may note that the Zhou et al. paragraph was written correctly to my intentions in the previous draft, referring to whole body. Again, I regret this presentation error and thank the reviewer for catching it.

In terms of the issue I raised of not stating which multiple tests were performed and correcting for it, instead of doing that the author declared the analysis an exploratory analysis. As the author acknowledged in the response the hypotheses selected to test were based on following previous significant analyses on the same data, thus because of that bias it would be challenging to formally correct for the multiple testing anyway.

I acknowledge the reviewer's agreement that formal correction for multiple tests would be challenging here. However, I want to clarify that the manuscript does state exactly which multiple tests were performed, helping to address the reviewer's original concern.

The author also presented a robustness analysis for the parameters, though that analysis was limited in that the selected parameter values were always one of the extreme values in the analysis. Additionally the analysis was shown for only the two slices of the grid of hyper-parameter values that had at least one selected value opposed to the entire grid. The author may want to consider expanding the robustness analysis with these considerations, but this is a minor point.

I have revised Supplementary Figure 13, which displays a parameter sweep over the gene distance threshold, to include an additional threshold of 30 kb, less than the chosen 50 kb. At this value, the score difference between probands and siblings grows relative to the larger thresholds, but the significance decreases due to the smaller number of variants remaining after the filter.

In Supplementary Figure 14, which displays a parameter sweep over the negative prediction weight, the first row displays the statistical tests computed for only negative values. This represents the limit as the negative prediction weight grows to infinity. Thus, it is a more extreme value than the chosen weight of 10x. I have clarified this in the figure and caption.

A comprehensive grid search over these parameters would require many dozens of additional combinations that I have not tested in my analysis and introduce a nontrivial visualization challenge. I do not think readers would derive sufficient value from this addition.

In terms of the issue of evaluating on additional human data, I agree with the author given the effort that the author says would be involved to investigate it, it is likely not worth the effort.

I appreciate the reviewer's thoughtful consideration of the benefits and costs in such an analysis.
